# Neural Stem Cell Niches in Health and Diseases

**DOI:** 10.2174/138161212799859611

**Published:** 2012-05

**Authors:** Ilaria Decimo, Francesco Bifari, Mauro Krampera, Guido Fumagalli

**Affiliations:** 1 Department of Public Health and Community Medicine, Section of Pharmacology, University of Verona, Italy; 2 Department of Medicine, Stem Cell Research Laboratory, Section of Hematology, University of Verona, Italy

**Keywords:** Neural stem cells, neuroblast, nestin, neurogenesis, neurotrophic factors, stem cell niches, meninges.

## Abstract

Presence of neural stem cells in adult mammalian brains, including human, has been clearly demonstrated by several studies. The functional significance of adult neurogenesis is slowly emerging as new data indicate the sensitivity of this event to several “every day” external stimuli such as physical activity, learning, enriched environment, aging, stress and drugs. In addition, neurogenesis appears to be instrumental for task performance involving complex cognitive functions.

Despite the growing body of evidence on the functional significance of NSC and despite the bulk of data concerning the molecular and cellular properties of NSCs and their niches, several critical questions are still open.

In this work we review the literature describing *i)* old and new sites where NSC niche have been found in the CNS; *ii)* the intrinsic factors regulating the NSC potential; *iii)* the extrinsic factors that form the niche microenvironment. Moreover, we analyse NSC niche activation in *iv)* physiological and *v)* pathological conditions.

Given the not static nature of NSCs that continuously change phenotype in response to environmental clues, a unique “identity card” for NSC identification is still lacking. Moreover, the multiple location of NSC niches that increase in diseases, leaves open the question of whether and how these structures communicate throughout long distance. We propose a model where all the NSC niches in the CNS may be connected in a functional network using the threads of the meningeal net as tracks.

## INTRODUCTION

The complex architecture of the adult brain is the product of genetic instruction, cellular cross-talk and interactions between the organism and the external world. The final result is an extensive network of hundreds of billions of neurons, in large part generated before birth, and many more glial cells.

Soon after gastrulation, the part of ectoderm immediately above the notochord specifies into the neuroectoderm that eventually proliferates and forms the neural tube. Cells of the neural tube, known as neural precursor cells, are dividing stem cells that symmetrically produce more precursors. The subventricular zone (SVZ) is site of an extraordinary mitotic activity and approximately 250,000 new neurons are generated each minute at the peak of cell proliferation during gestation.

Newborn neurons derive from less differentiated neural stem/progenitor cells (NSCs). The concept of NSC originates from studies on the development of the central nervous system (CNS) [1]. During development the neuroectoderm forms the earliest pluripotent NSCs, called neuroepithelial cells (NEPs), which then further differentiate into neuronal-restricted or glial-restricted precursor cells [1]. Human NEPs can be isolated from foetus and from embryonic stem cells [2, 3]. These cells form neural rosettes and express nestin, a neural intermediate filament protein, and musashi-1, a neural RNA binding protein [3]. Nestin is recognized as the most relevant marker of neuroepithelial cells [2, 3]. *In vivo* expression studies in mouse and chicken indicate that undifferentiated NEPs specifically express the transcription factors Sox1, Sox2 and Sox3 [5, 6].

Up to a few years ago it was believed that neurogenesis was temporally restricted to the embryonic life and that new neurons are not generated after birth. Thanks to pioneering work done in the late sixties [7], this view has changed. It is now widely accepted that new neurons are continually generated throughout the life and that they become functionally integrated into the brain tissue. The functional significance and role of adult neurogenesis is under extensive investigation and new data are accumulating. Many experience-related and environmental clues have been shown to regulate neurogenesis. Even more strikingly, reactions to these clues appear to be partially determined by neurogenesis. Adult neurogenesis has also been reported in humans [8].

On the other hand, adult neurogenesis is not a diffuse event and apparently occurs in restricted regions, where classical developmental signals and morphogens like Notch, Bone morphogenic Proteins [BMPs), Eph/ephrins, Noggin, and Sonic hedgehog homolog (Shh) are maintained [9]. Of these sites, the best described are the SVZ of the lateral ventricle wall and the dentate gyrus subgranular zone (SGZ) of the hippocampus [10]. Neurogenesis may also occur in other brain areas, including substantia nigra [11], striatum, amygdala [12] and neocortex [13] and several new potential sites of neurogenesis have been described in the recent years. So far, a comprehensive map of neurogenic sites of the brain has been drawn for songbirds only [14]. In other species the question of the origin of the newly formed neural cells as well the hunt for the identification of new sites of neurogenesis are still open. It is therefore relevant to define common criteria for definition of NSC and of their hosting sites, also called niches.

### Operational Criteria for NSC and their Niche Definition 

Although many progresses have been made in the understanding of NSC biology, the identity of NSCs and the factors regulating their fate are not fully understood. In particular great efforts have been made to unequivocally identify the primitive, more immature, quiescent NSCs and to distinguish them from the amplifying and differentiating cell pools. Up to now, a unique identification criterion of NSC is lacking, thus leading to contradictory results on the distribution and features of resident NSCs and on their differentiation potential *in vivo*.

Definition of stemness relies on both *in vivo* and *in vitro* criteria. 

The neurosphere assay is the most commonly accepted criterion for *in vitro* demonstration of NSCs. The method [15] has allowed identification of putative NSCs in many areas of adult mouse brain. Isolated adult SVZ-NSCs proliferate in the presence of the mitogenic epidermal growth factor (EGF) and form neurospheres (50-150 μm in diameter) of proliferating undifferentiated neural cells [15]. These can be either serially transferred to expand the amount of neurospheres or differentiated both *in vitro* and *in vivo* into neuronal and glial cells [16]. These results demonstrate the two functional attributes that define stem cells: self-renewal and multipotency. Nowadays, neurospheres cultures can be obtained from many regions of the brain, including olfactory bulb, cerebellum, white matter [17,18], spinal cord [19], substantia nigra [20], retina [21], hypothalamus [22], hypophysis [23].

Identification of NSCs *in vivo* in adult brain has traditionally relied on analysis of cell morphology, mitotic activity and protein and gene expression.

The comparative ultrastructural analysis of the ventricular proliferative zone in different species has allowed identification of different cell types in this region [24]. The presence of ultrastructurally distinguishable cells and their spatial relationship within the subventricular region [25] and the specific morphological organization of the vasculature in this region have also been described in details [26]. Most commonly, analysis of the different cell pools relies on identification of specific markers, several of which are developmentally retained.

Nestin [27], Glial Fibrillary Acidic Protein (GFAP) [28], Musashi 1 and 2 [29], Sry-related high mobility group box transcription factor SOX2 [30] are some of the most commonly used NSC markers. Nestin is a class VI intermediate filament protein expressed in mitotically active areas of the developing and adult brain [31]. GFAP is an intermediate filament protein expressed by astrocytes and several other cell types, including ependymal cells and radial glia. Its expression in (some but not all) NSCs is considered remnant of the radial glia origin of the cell [28]. Musashi 1 and 2 were first found to be expressed by neuroepithelial cells in the embryonic ventricular zone. Sox2 is a transcription factor essential to maintain self-renewal properties of undifferentiated embryonic stem cells. Any of the above markers should not be use as unique criterion for NSC identification.

Definition of the state of the NSC is also based on expression of specific markers. 

Quiescent stem cells are recognized by the expression of Oct4, Sox2, Nanog, FoxO3 [31, 32, 33], as well as on long-lasting retention of bromodeoxyuridine (5-bromo-2'-deoxyuridine, BrdU), a thymidine analogue [24, 34]. This assay is based on the concept that BrdU becomes incorporated in the DNA if a cell proliferates during exposure to the tracer (from a few hours up to few days). The staining persists if the cell remains quiescent, whereas it declines in mitotically active cells (dilution effect). The presence of BrdU-positive cells 3-4 weeks after BrdU administration is an indication of quiescence.

The transient-amplifying cells can be studied by analysis of the expression of cell-cycle specific markers and of incorporation BrdU or 3H-thymidine. Markers of cell-cycle entry, Mcm-2 and Ki67, and of cell-cycle phases including cyclins D1 and E (markers of G1 phase), cyclin A (S phase), cytoplasmic cyclin B1 (G2 phase), and phosphohistone H3 (M phase) have been used [35].

Differentiated precursors are commonly recognized by the expression of doublecortin (DCX) and of the polysialylated neural adhesion molecule PSA-NCAM [36, 37]. Fate analysis of BrdU-labelled cells has also been commonly used to characterize the differentiation potential of NSCs. Electrophysiological approaches have been used to define the functional properties *in vivo* of these differentiated precursors [38, 39]. 

Along with the classical molecular biology and morpho-biochemical approaches, in recent years the use of transgenic mice has been applied for a more precise *in vivo* stem and progenitor cell mapping. The common strategy in this approach has been the introduction of a reporter gene under the control of genes expressed by specific cells in the adult NSC lineage. Interestingly, for each candidate markers, such as nestin, many different transgenic mouse strains have been created. Of note is that the distribution profile of the transgenic protein expression (usually GFP, green fluorescent protein) may not always match the endogenous protein. Indeed, the expression appears to vary among animal lines with different transgene constructs [40,41]. These discrepancies are mainly depending on which part of the gene regulatory elements (promoter/enhancer) are controlling the expression of the reporter gene.

An incredible expansion in NSC biology has been given by the use of inducible transgenic mice. With this model it is possible to identify and study the distribution of cells primed for a specific protein in a defined time-window. This approach allows fate analysis of NSCs and makes feasible studies revealing experience- or pharmacological-dependent activation and differentiation of NSC. By combining old and new methods, i.e. observing the population of genetically labelled cells at different time points after pulse BrdU labelling, it is possible to get insights on the identity, proliferation and fate of new born cells.

Regardless of their origin, NSC reside in specific sites called niches. A niche provides conditions for maintenance of the stem cell pools in a quiescent state as well as signals for activation and differentiation when neurogenesis is required. NSCs and their niche form a functional as well physical unit endowed of specific, and sometimes unique, molecular properties. Understanding the composition, the function and the nature of the signals exchanged in the niche may open the stage to a therapeutic exploitation of the NSC potential of different areas of the brain. 

This review will summarize the current knowledge on the molecular, structural and functional properties of NSCs and their hosting niches. Cellular and microenvironmental factors defining a quiescent NSC niche and changes induced by physiological, pharmacological and pathological stimuli will be reviewed with the goal of defining a conceptual frame for NSC niche identification and modulation of function. The distribution and the differences among NSCs in different niches will be analysed, and a model for integrating different and distant niches into a unique functional network will be proposed. 

## NEURAL STEM CELL NICHES IN ADULT CNS

A niche is made up by set(s) of cells including the stem cells whose state (quiescence, self-renewal, amplification or differentiation) is determined by a combination of cell specific properties (intrinsic determinants) and of signals residing and/or spreading in the microenvironment hosting the stem cells (extrinsic determinants).

Intrinsic determinants are mainly related to the epigenetic status of the stem cells and to their molecular repertoire required to sense the complex net of extrinsic signals operating in the niche. Extrinsic determinants include extracellular signals/factors, such as growth factors, cell-to-cell and cell-to-ECM contacts. They regulate embryonic and adult stem cell biology; they also regulate the behaviour of the cells during development and in adults and their responses to physiological and pathological stimuli [42].

In this part of the review we will first review data concerning location of the niches that have been so far identified in adult CNS and the intrinsic and extrinsic determinants that act to confer to resident cells their stemness-related properties. Two major aspects will emerge from the review of these aspects: 1- NSC-containing niches are copious and, with a single exception, minute; 2- factors determining/controlling stemness and its progression are multiple.

### Location of NSC Niches in Adult CNS

In the early embryonic stages NSCs line between the ventricular surface and the pia mater and generate *in vivo* to the whole cortex and spinal cord. These developmental features are apparently retained in the ventricular/periventricular zones also in adult brain. For this reason the ventricular/periventricular zones have been extensively studied in the last decades and many of the features characterizing the microenvironment of this NSC niches have been revealed.

NSC niches outside of the “classical” neurogenic region have also been identified. It must be pointed out that the major and ultimate criterion for identification of a niche is the presence of NSCs. Accordingly, NSCs have been found in the sub-granular layer of the dentate gyrus of the hippocampus and, more recently, in several other regions of the CNS. However, in some cases the characterization of the NSCs present in these niches has not been complete and it is not matching all the strict criteria that should be used to define classical NSC (i.e. *in vivo* assessment of self renewal, proliferation and multipotent neural differentiation). Despite this limitation, CNS regions containing high density of NSCs or any of the components of the NSC pools (quiescent and/or amplifying and/or neural precursors) have been described. At these sites these cell populations persist and appear to be sensitive to external clues. Example of distribution of putative NSCs are provided in Figs. (**1**, **2** and **3**) where antibodies against the three relevant NSC markers GFAP, Sox2 and nestin have been used.

Insights of the mechanisms by which NSC maintenance, expansion and differentiation are regulated within a niche may provide the knowledge required to identify new pharmacological targets and tools for the cure of several neurologic diseases. In addition, a comprehensive view of the distribution of NSC niches in CNS may give additional clues for understanding NSCs biology. Finally, the possible integration of different niches into a unique network may provide a new conceptual frame for considering the functional significance of NSCs for CNS homeostasis and repair.

#### Subventricular Zone

This niche has been extensively studied. The SVZ is considered a germinal zone remnant persisting in adulthood and continuously supporting the olfactory bulbs [43, 44] and the corpus callosum [45] of new cells. Different cell types have been identified by morphological ultrastructural criteria: quiescent NSCs, migrating neuroblasts, astrocytes with lax or dense chromatin, amplifying precursors, tanycytes and ependymal cells. Quiescent NSCs appear to have irregular cell profiles closely apposed to neighbouring cells, a light cytoplasm with few ribosomes, extensive intermediate filaments and invaginated nuclei. Neuroblasts have an elongated cell body, microtubules oriented along the long axis of the cells, dark cytoplasm with abundant ribosomes and occasionally invaginated nuclei with small nucleoli. Transient amplifying neural progenitors are large and semi-spherical, their nuclei contain lax chromatin with large nucleoli and their cytoplasm shows few ribosomes and no intermediate filaments. Both tanycytes and ependymal cells face into the ventricular space with an apical membrane endowed of microvilli and cilia [24].

First attempts of NSC identification in SVZ suggested that ependymal cells were the adult NSCs responsible for neurogenesis in this region [46, 47]. Administration of BrdU to adult mice for 2 weeks labelled many ependymal and SVZ cells but ependymal cells only displayed long-lasting BrdU retention (a criterion for quiescent NSC identification) [47]. Based on the same criterion, it has been proposed that tanycytes in the third ventricle [48] and possibly in the adult spinal cord [49] may also be NSCs. On the other hand, other studies have shown that ependymal cells are quiescent and do not have the properties of NSCs [50, 51]. Indeed, studies with adenovirus expressing green fluorescent protein (GFP) under the GFAP promoter suggested that SVZ astrocytes are NSC candidates [51, 52]. This was supported by the observation that GFAP-expressing cells labelled *in vivo* in the SVZ generate cells that migrate to the olfactory bulb and differentiate into neurons. Finally, the use of lox-CRE-based techniques has allowed postulating that adult periventricular NSCs derive from radial glia [53]. Using immunohistochemistry and confocal microscopy on the whole mount preparations of the ventricular surface it was found that both SVZ-astrocytes (with one cilium) together with ependymal cells (with two or multiple cilia) contacted the ventricular surface. Given the many similarities between SVZ astrocytes and tanycytes, it has been proposed that they may derive from a common ancestral progenitor [54].

*In vivo* SVZ precursors generate primarily committed neuronal precursors that migrate tangentially along the rostral extension of the SVZ toward the olfactory bulb, constituting the rostral migratory stream [44] After reaching the olfactory bulb neuronal precursors move radially into the granular and periglomerular layers, where they differentiate into mature neurons [55, 56].

Migration of SVZ cells to other regions of the CNS also occurs in pathological condition. For example, brain injury is associated to production of neuroblasts that migrate from SVZ into the neighbouring striatum [57]. SVZ-derived new neurons have also been described to migrate, survive and mature within areas of stroke [58, 59]

The functional diversities of SVZ precursor cells is underlined by the expression of several different NSC markers, such as GFAP, Sox2 and nestin Figs. (**1**, **2** and **3**). It must be pointed out that these three markers can be expressed in a single NSC; more important is that none of them can independently identify NSCs in SVZ. Indeed, the presence of NSCs in different quiescent-proliferating-differentiating stages could reflect this heterogeneity of phenotypes and may contribute to create the complex cytoarchitecture of the SVZ [24]. 

#### Subgranular Zone

In the subgranular zone (SGZ) two types of neural progenitors have been identified based on their morphology and the expression of specific molecular markers. Type 1 hippocampal progenitors have a radial process spanning the entire granule cell layer and ramifying in the inner molecular layer. These cells express nestin, GFAP and Sox2 [60, 61]. Although they express the astrocyte marker GFAP, these cells are morphologically and functionally different from mature astrocytes. Type 2 hippocampal progenitors have only short processes and do not express GFAP. Type 2 cells may arise from type 1 cells, but direct evidence proving this relationship is still lacking. Suh *et al* provided the first *in vivo* evidence that type 2/Sox2-positive cells can self-renew and give rise to neurons and astrocytes [62].

#### Hypothalamus and Circumventricular Organs

Recent evidence indicates that the hypothalamus hosts a neural stem cell niche at the level of the region lining the third ventricle [63, 64]. Numerous reports in different species have consistently provided evidence of the existence of constitutive neurogenesis in the adult hypothalamus [63-66].

Recently, a detailed description of the CVO (circumventricular organs) as niche for neural stem cell has been provided [67]. The proposal is based on expression in this region of markers like nestin, GFAP and Sox-2 in cells also expressing the proliferative marker Ki-67 and loaded with BrdU. By using nestin-GFP transgenic mouse, the authors showed that nestin-positive cells extracted from this region can generate neurosphere *in vitro* giving rise to glial cells and immature neurons.

This region also shares common features with the SVZ at the level of extracellular matrix composition and ultrastructural organization. ECM extensions, named fractones, consisting of labyrinthine basal lamina, were shown to project from blood vessels of the subependymal layer to terminate immediately beneath the ependyma [68]. Fractones have also been described at the level of SVZ [69]. 

#### Cerebral Cortex

Cells with some of the NSCs properties have been found in adult cerebral cortex and indication that this niche may contribute to neurogenesis has been provided [70]. In particular, A2B5-positive glia-restricted progenitor and NG2-positive cells have been described as source for *in vitro* NSC expansion, also when derived from human adult brain. The A2B5-positive cells are glia-restricted progenitor (GRP) cells able to differentiate into oligodendrocytes, type-1 and type-2 astrocytes. Sorted A2B5-positive cells from the adult subcortical white matter of the adult human brain generate multipotent neurospheres in culture that exhibited site-specific multipotent differentiation potential following *in vivo* transplantation [71]. This type of cells is of interest as SVZ-derived GFAP (low)/A2B5+/nestin+ have been identified as candidate founder cells capable to generate large numbers of fully differentiated interneuron phenotypes *in vitro* [63].

NG2-positive polydendrocytes are defined as CNS parenchymal cells (non-vascular cells) that express the integral membrane chondroitin sulphate proteoglycan (CSPG4), also known as NG2. These cells are oligodendrocyte progenitor cells (OPCs) that generate oligodendrocytes during development and in adult CNS. OPCs express platelet-derived growth factor receptor α (PDGFRα) whose stimulation increases cell survival and proliferation. Fate mapping of OPCs using NG2-Cre-transgenic mice showed that these cells also differentiate into a subset of protoplasmic astrocytes in the grey matter [72].

NG2-positive cells have also been proposed to originate the SVZ transient-amplifying cells that contribute to interneuron neurogenesis in the postnatal hippocampus [73, 74]. Fate map studies using PDGFRα-CreERtransgenic mice carefully demonstrated that OPCs in the adult brain generate a small number of neurons in the piriform cortex but not in the olfactory bulb [75].

The origin, function and fate of neuroblasts found in the CNS parenchyma are still obscure; in addition it is not clear whether a *sensu stricto* niche is present to host them [76-79]. 

In cortical layer I of the adult rat neocortex a small number of dividing cells (largely ignored until recently) have been described in the subpial zone. These cells have some characteristics of interneurons but not of glia (they express the GABA-synthetic enzyme GAD67 but not other neuronal markers). The fates of these cells have been traced by injecting retroviruses into the subpial zone; it was shown that they do not originate new neurons in the brain under physiological conditions [80].

During these years, different experimental approaches have been used to identify immature neurons in cortical layer II, to determine their time of generation and to characterize their phenotype and fate. These approaches include the analysis of immature and mature neuronal markers, cell birth dating, structural and ultrastructural studies and functional analyses [81]. As result, immature neurons identified by the expression of PSA-NCAM and DCX have been found in the cortical layer II of the cortex across different species [81, 82]. These cells lack the expression of the neuronal mature marker NeuN [36, 37, 83-85] and of glial markers [36, 84, 85]. The origin of these immature neurons is not clear and it has been suggested that they may have embryonic origin [84, 86]. Their fate in cortical layer II is still not clear and it has been proposed that they may migrate since these cells disappear from cortical layer II during aging without leaving behind traces of apoptosis [81]

#### Cerebellum

The cerebellum rabbit shows remarkable neurogenesis around puberty [87] that persists to a lesser extent during adulthood [77].

The proliferating elements are in the subpial layer where they form a single, non-continuous layer independent from the meninges [87]. Uptake of systemically-administered BrdU has been used to show that a substantial amount of these cells is still alive two weeks after their birth [87], and to a lesser extent, after two months [77]. The newly generated cortical cells fall into three main morphological types: bipolar, polarized neuronal-like and multipolar that can be further divided in two populations: neuroblast neural precursor (positive for DCX-PSA.NCAM-Pax2] and glia-like cells (positive for MAP5-olig2-sox2] [77, 79]*.*

The dynamics of the neurogenic process in the peripuberal and adult rabbit cerebellum are remarkably different from those described in other mammalian species studied so far. The production of new cell progenitors, including neuronal precursors, continues at high rates up to and beyond puberty, then progressively decreases with age [77, 87].

#### Olfactory Bulb and Mucosa

The majority of the lateral ventricle-derived cells that are migrating along the rostral migratory stream (RMS) into the olfactory bulb are non-proliferating although mitotically active precursors within the RMS have also been described [88] NSCs can be cultured from all parts of the rostral extension, including the region within the olfactory bulb. Of note is that these *in situ* generated precursors reside within the rostral extension and do not migrate from the SVZ [89]

Despite the high rate of proliferation of the neuronal precursors, the thickness of the olfactory epithelium remains stable in the adult rat from 60-330 days of age [90, 91]. Indeed, adult neurogenesis in the olfactory epithelium is tightly regulated and the number of mature sensory neurons is maintained to provide a constant surface density of sensory dendrites [92]. Interestingly, there is a large overproduction of immature neurons with only a subset of cells that survives [93-95]. Apoptosis occurs at all stages of olfactory neuron development from recently born basal cells to immature and mature neurons [96-99]. 

Neurogenesis is stimulated by the death of the sensory neurons induced by transection of the olfactory nerve [100] and by toxins like zinc sulphate and methimazole [101, 102] which cause apoptotic cell death [96, 103, 104]. The loss of neurons increases basal cell mitosis [100, 105] with recovery of the sensory neuron population [106, 107], of function [108, 109] and of the sense of smell [109, 110].

The weight of evidence indicates that the horizontal basal cell is the stem cell responsible for regeneration. The horizontal basal cell proliferates slowly and self-renews [94] and it generates all the olfactory epithelial cell types both *in vivo *and *in vitro *[111, 112]. Chemical ablation of the olfactory epithelium induces proliferation of the horizontal basal cells and subsequent regeneration of the epithelium and recovery of the smell function [113]. Reconstitution of the olfactory epithelium after chemical ablation is also achieved by transplantation of globose basal cells which, like horizontal basal cells, give rise to sensory neurons, supporting cells and Bowman’s glands and duct cells [99, 100]. Because the globose basal cell arises from the horizontal basal cell *in vivo* [112], it is likely that the horizontal basal cell is the “true” tissue stem cell that maintains long-term regenerative capacity of the olfactory epithelium, whereas the globose basal cell is a multipotent, transit amplifying cell and immediate neuronal precursor [114, 115].

The horizontal basal cells express ICAM-1 and the integrins β1, β4, α1, α3 and α6 which act as receptors for collagens, fibronectin and laminin in the basement membrane [111]. When selected for ICAM-1 expression and grown as clones, these horizontal basal cells demonstrated the stem cell property of self-renewal and were capable of differentiating into globose basal cells, olfactory sensory neurons and glia [111]. Although not yet identified positively as a particular cell type, olfactory stem cells have been grown *in vitro* from biopsies of adult mouse, rat and human olfactory mucosa. This cell is multipotent and can generate cells not only of the epithelial and neural lineages but also of the embryonic mesodermal and endodermal lineages [116, 117].

The complexity of the neurogenic niche of the olfactory epithelium is illustrated by the very large number of growth factors and their receptors present in the olfactory epithelium [118]. For example neurotrophins and their receptors are each expressed by specific sets of cells [119]. Horizontal basal cells express TrkA and NT4 whereas sensory neurons express TrkB and TrkC and all the neurotrophins [119]. Supporting cells, developing neurons and olfactory unsheathing cells express other combinations [119]. The horizontal basal cell expresses the epidermal growth factor receptor, EGFR and proliferates in response to its ligands EGF and Tiff [115]. 

The olfactory mucosa is accessible in living adult humans and is therefore a source of tissue useful for studying the biology of adult neurogenesis in health and disease [120]. It is also a source of cells for transplantation (regenerative medicine) of the nervous system [121, 122]. Understanding the biology of olfactory neural stem cells and their niche will be very important for optimizing clinical applications.

#### Retina

The retina completes its development early after birth and no additional retinal cells are produced thereafter. Based on this feature and on the absence of tissue repair following damage, the adult mammalian eye was considered devoid of retinal stem cells (RSCs). In 2000, however, two teams independently demonstrated that single pigmented cells from the ciliary epithelium of mouse retina could clonally proliferate *in vitro *to form sphere colonies [21, 123]. A small number of pigmented cells from these primary colonies repeatedly generated new secondary spheres indicating that the initial colony-forming cells had the capacity to self-renew. In addition, exposed to differentiation conditions, the colony forming cells were shown to express genes found in rod photoreceptors, bipolar neurons, and Müller glia, suggesting their multipotentiality. The idea was thus born that the ciliary body of the adult mammalian eye harbours a population of retinal stem cells, present in a mitotically quiescent state *in vivo*. A few years later, these cells were identified in the ciliary bodies of other mammalian species, including human [124-127].

The molecular signature of these cells has also been defined. Among the markers are the paired-class transcription factors, Rx, Chx10 and Pax6, the homeodomain-containing transcription factors, Six6 and Six3 or the LIM homeodomain factor Lhx2 [123, 127-131]. Comparative transcriptional profiling revealed that ciliary body-derived cells and embryonic retinal progenitors share 80% identity of their expressed genes [128-130]. In addition, Pax6, Rx, Chx10 and Six3 were found to be expressed within the ciliary epithelium of adult rodents, monkeys and humans consistent with the presence of retinal stem/progenitor cells *in vivo* [130, 132]. Finally, recent findings unexpectedly suggested that in physiological conditions, ciliary body stem cells might contribute to retinal cell turnover of adult primates [132]. Müller glial cells may also represent a potential source of stem cells within the neural retina of the mammalian eye, although this aspect will not be reviewed here [133, 134].

The eyes of fish and frogs have a characteristic small peripheral zone at the junction between the ciliary epithelium and the retina, that is mitotically active and it is called ciliary marginal zone (CMZ) [135, 136]. The possibility that an active CMZ-like region exists in the adult mammalian eye is currently an area of growing research interest [137, 138]. Indeed, reports of the expression of nestin by cells present in the most peripheral region of primate and human retina, suggest the presence of progenitor cells in these species [139, 140]. These cells also express the Müller glial cell marker CRALBP as well as other markers of neural retinal stem/progenitor cells, including CHX10, Sox2 and SHH [139]. 

#### Spinal Cord

Little is known about the *in situ* localization, activity, regulation, and function of adult spinal cord NSCs. Two models have been proposed regarding the location of these cells in the intact adult spinal cord: in the first, a slowly proliferating stem cell resides in the ependymal layer of the central canal [47]; in the second, stem cells and glial progenitors are suggested to exist in the parenchyma of the spinal cord [141] and to be independent of the proliferative ependyma. 

*In vitro* NSCs have been cultured from the ependymal zone surrounding the central canal of the spinal cord. The spinal cord ependymal cell niche has been fully described for the first time by Hamilton and colleagues [142]. They observed that central canal is lined with ciliated ependymal cells expressing vimentin. Ki67-positive proliferating cells are primarily found within the ependymal zone and are distributed in a dorsal to ventral gradient. Proliferating cells are found in doublets and are always closely opposed to blood vessels. There are nestin-expressing and GFAP-expressing vimentin-positive cells at the dorsal pole that possess extensive basal projections. Within the sub-ependymal layer, there are GFAP positive astrocytes, NeuN-positive neurons and Olig2-positive oligodendrocyte progenitors. Spinal cord ependymal cells have neural stem cell potential both *in vitro* and, following tissue injury, *in vivo*. Cells coexpressing PSA-NCAM and DCX and contacting the central canal of the spinal cord have been described in neonatal rats; these cells lack mature neuronal markers and show ultrastructural and electrophysiological features of immature neurons [143].

Neuroblasts have been identified in adult spinal cord parenchyma as well [144, 145]. Here, the progenitor cells give rise to immature neurons expressing DCX, GAD-65/67, and GABA [145]. The presence of proliferating cells within the dorsal part of the spinal cord suggests that the newly formed GABAergic neurons in the adult spinal cord arise from a progenitor-cell population different from that of the central canal, in accordance with the model proposed by Horner *et al*. [146]. Interestingly, cell transplantation studies have demonstrated that, although NSCs derived from spinal cord will differentiate into glial cells when implanted into the region of origin, they are able to give rise to neurons when heterotopically grafted into the neurogenic hippocampus [147]. Whether this is a property of this cell population or is a consequence of specific local signals acting on any migrating NSC or a combination of both remains to be determined.

In non pathological conditions, ependymal cell and astrocyte self-renew to maintain their pool, whereas oligodendrocyte progenitors self-renew and give rise to an increasing number of mature oligodendrocytes [148]. Glial progenitors have been described in the outer circumference of the spinal cord and they can give rise to both astrocytes and oligodendrocytes [146]. 

Spinal cord injury recruits ependymal cells, astrocytes, and oligodendrocyte progenitors to generate progenies; most of the ependymal progeny becomes astrocytes, it modestly contributes to oligodendrocyte generation and does not participate to neuron generation [148]. 

#### Meninges

Meninges are a system of membranes that envelop the CNS (brain and spinal cord) consisting of three layers: dura mater, arachnoid and pia mater. Dura mater is attached to the skull or to the bones of the vertebral canal in the spinal cord. Taken together, the arachnoid and pia mater are called the leptomeninges and contain the cerebrospinal fluid (CSF), trabecular cells, arteries and veins. Meninges were considered as an anti-shock device protecting the brain from traumas and to be devoid of any functional connection with the brain parenchyma. Indeed, a continuous mat of extracellular matrix is localized at the surface of the brain as well as around the blood vessels in the brain. This material is considered to form a sharp interface separating (both anatomically and functionally) the brain parenchyma (neurons and glia) from the extraparenchymal tissues (meninges and vessels). 

More in depth studies on the function and ultrastructure of meninges have changed this view. Leptomeninges form a complex microenvironment that has important functions for the normal cortex development [149]. They are present since the very early embryonic stages of cortical development, when columnar neuroepithelium is located between the ventricle surface and the pia basal membrane. Leptomeninges are site of in multiple interactions involving a large number of molecular and chemotactic factors (e.g. SDF-1/CXCR4, reelin, oxidative state) [150-152], cell types (e.g. pia mater cells, radial glia, neural precursor cells, Cajal Retzius cells, glia limitans cells) [153, 154] and extracellular matrices components (e.g. laminin, collagen IV, fibronectin) [155-157] that ensure correct cortical development. Abnormal function/structure of leptomeninges causes altered cortical histogenesis, as in the case of cobblestone lissencephaly (type II), where the fragmentation of pia mater basal membrane leads to the formation of cortical neurons protruding into the sub-arachnoid space [158]. In adults meninges are endowed of several trophic factors, including FGF-2 [159], CXCL12 [160] and retinoic acid [161].

Meninges also have a relevant intraparenchymal distribution. Every parenchymal vessel inside the CNS is surrounded by a perivascular space (Virchow-Robin space) formed by the extroflexions of leptomeninges (arachnoid and pia mater) filled with CSF. Mercier and Hatton fully described the distribution and the connection between meninges and vessels providing evidence that meningeal cells are distributed and abundant also inside the CNS parenchyma [162]. Cells of the meninges, of the choroid plexus and of the perivascular structures form a functional network [68, 159, 162]. Within this network comprising parenchymal astrocytes, meningeal cells express connexin 26 [159], a constituent of the gap junctions, thus providing a rapid mean to spread signals. A reconsideration of meningeal functions is thus a must.

A new perspective has been opened by our discovery that meninges (arachnoid and pia mater) host NSCs [163]. In that work, we have analyzed the leptomeningeal compartment of the rat brain to assess whether a stem cell population with neuronal differentiation potential may be present in this structure. Indeed, we found that (i) nestin-positive cells were present in the leptomeningeal compartment at the embryonic stages and persisted up to adulthood, (ii) leptomeningeal nestin-positive cells could be extracted and cultured as neurospheres with features similar to the NSC-derived neurospheres, (iii) leptomeningeal nestin-positive cells could also be cultured as adherent cells and expanded *in vitro* as homogeneous population of nestin-positive cells that highly express many of the stemness-related genes, (iv) expanded nestin-positive cells could be induced to differentiate *in vitro* with high efficiency to generate excitable neurons and (v) expanded cells differentiated into neurons when injected into brains of living rats. 

Our observations were in agreement with previous indication that NSCs were present in the choroid plexus of adult rat [164] and that cells from human meninges express some neural markers when cultured *in vitro* [165]. Nestin-positive cells have been shown to be present also in human meninges [166, our unpublished data from adult human biopsies]. 

More recently, we also observed that nestin-positive and nestin/DCX-double positive cells are present in the meninges of the spinal cord of the adult rat [167]. Following a moderate contusive spinal cord injury (SCI), the meningeal nestin-positive cells proliferate and increase in number; in addition, nestin-negative/DCX-positive cells appear, suggesting a progression toward the neural fate. We used lentiviral transduction of meninges to show that meningeal nestin- and DCX-positive cells contribute to glial scar formation after SCI, giving a new insight into the complexity of the parenchymal reaction to a traumatic injury [167]. 

These findings indicate that meninges share common properties with classical NSC niches including the presence of cells with neural precursor features. The origin of these cells has not been determined yet; on the other hand, we also showed that meninges can host heterologous NSCs injected into the ventriculum [Fumagalli G., Decimo I., Bifari F., Krampera M. Homing and migration of transplanted Leptomeningeal Stem/progenitor Cells in adult rat brain. Program No. 34.12/E16. 2010 Neuroscience Meeting Planner. San Diego, CA: Society for Neuroscience, 2010. Online]. Altogether the data suggest that meninges are functional NSC niches. Meninges are also more accessible than other neural stem cell niches, an aspect that has interesting implication for collection of NSCs for regenerative medicine.

### Intrinsic Factors

“What does it control where and how adult neurogenesis occurs?” The answer to this question is complex and both “cell-intrinsic molecules” and external signals produced in the microenvironment should be advocated.

Intrinsic factors are part of the exceptionally tight control of neurogenesis, regulating proliferation and differentiation. Despite the technological advances, analysis of intrinsic pathways that regulate adult neurogenesis have not been thoroughly studied also because many traditional constitutive knockout mice are not viable. In this review we will focus on the receptors and on the epigenetic events that confer to NSCs their unique properties.

#### Receptors

##### Notch

The Notch signalling network is an evolutionarily conserved intercellular signalling pathway which regulates interactions between physically adjacent cells. Notch1 is a membrane receptor whose full length form is cleaved in the transGolgi network to generate the membrane heterodimeric form [168]. Membrane-bound Notch binds to ligands (e.g. Delta) present on an adjacent cell. When a gamma secretase cleaves the membrane bound Notch receptor, the Notch intracellular domain (NICD) is translocated to the nucleus [169] where it interacts with mastermind-like (mam1). Mam-1 converts the required transcriptional cofactor of Notch, RBP-J, from a transcriptional repressor to an activator. Transcription of downstream target genes, including Hes1 and Hes5, is then activated [170, 171]. 

In neural stem cells, targets of Notch signalling work together to prevent terminal differentiation and preserve a pool of stem cells [172, 173]; Notch1 also promotes radial glia-like identity and negatively regulates cell cycle exit and neuronal differentiation in GFAP-NSCs in the postnatal brain [174]. The role of Notch1 in adult neurogenesis has not been clarified. Evidence of the role of Notch1 signalling pathway in the maintenance of the reservoir of stem cells in the adult has been shown by Ables *et al*. [175]. Using nestin-CreER T2/R26R-YFP/Notch1loxP/loxP [Notch1 inducible knock-out (iKO)] mice they provided evidence that Notch1 is required for maintenance of adult hippocampal stem and progenitor cells and of adult neurogenesis in hippocampus. In absence of Notch1, self-renewal and expansion of nestin-expressing cells was disrupted and the net number of adult-generated dentate gyrus neurons was decreased. 

Notch signalling maintains neural stem cell features of subventricular zone astrocytes, and its block results in the loss of these cells and precocious neurogenesis [176]. Ablation of canonical Notch signalling after genetic deletion of Rbpj, a key mediator of canonical signalling by all Notch receptors [177], induces ependymal cell generation by astrocytes. 

##### Ephrin-B/EphB Signalling 

Eph tyrosine kinase receptors and their ephrin ligands control cell-cell interactions in many developing and adult tissues [178]; they have been identified as important regulators of proliferation, differentiation, survival and migration of stem/progenitor cells [179]. Several Eph tyrosine kinase receptors and their ephrin ligands are expressed in the adult SVZ and are involved in regulation of the migration of neuroblasts and of the proliferation of neural stem/progenitor cells [180, 181]. Blocking the ephrin-B/EphB interaction by infusion of soluble ectodomains of ligands or receptors into the lateral ventricles results in remodelling of the niche in a way that is comparable to the effects of lesion or aging [180], providing an indication that this class of molecules may regulate niche cell plasticity. By combining genetic fate mapping with EphB receptor blockade, the Frisen group has recently provided evidence that EphB signalling controls lineage plasticity of adult neural stem cell niche [182]. 

EphB receptors are expressed in the lateral ventricular wall by ependymal cells and astrocytes [180, 183]. The blockade of EphB/ephrin-B signalling resulted in a cell fate shift between ependymal and astrocytic phenotypes. Furthermore, the same authors showed that EphB activity acts downstream of Notch signalling and is sufficient to rescue ependymal cell loss induced by a suppressed Notch signalling [182].

Constitutive Notch signalling is required for ependymal cell maintenance in the adult forebrain [46]; EphB2, that acts downstream of Notch, is also required for the maintenance of ependymal cell characteristics, by inhibiting the transition from ependymal cell to astrocyte. EphB2 overexpression is sufficient to rescue the effect of loss of canonical Notch signalling on ependymal phenotype [182].

##### Neurotrophin Receptors

Neurotrophins, such as brain derived neurotrophic factor (BDNF) and nerve growth factor (NGF), and their membrane bound receptors, TrkA, TrkB , TrkC and p75, are obvious candidates for regulation of neurogenesis, and direct demonstration of the selective functions of the different receptors have been recently provided [184]. NSCs express the receptors for the neurotrophins to various degree based on cell distribution and stage. Li *et al*. used a Nestin-CreERT2 system to genetically remove floxed TrkB from stem cells and their progeny [185]. They showed that loss of TrkB did not disrupt basal neurogenesis, but the mice failed to show an increase in neurogenesis upon administration of antidepressants, a common treatment to activate proliferation in hippocampus [186].

A second study by Bergami *et al*. utilized the GLASTCreERT2 system to ablate TrkB signalling in adult neural stem cells and their progeny [187]. Although GLAST is known to drive gene expression in astrocytes as well as stem cells, this group showed a decrease in survival of neurons after tamoxifen-induced deletion of TrkB.

Together these studies demonstrate the requirement of TrkB signalling in SGZ neurogenesis and hippocampus function. Specifically, TrkB appears to be important in neuroblasts for proliferation, integration and survival. The role of TrkB and other neurotrophin receptors in other NSC niches is less clear. These receptors are expressed in SVZ [188], olfactory mucosa [119] and meninges (our unpublished data) suggesting that they may have a role as intrinsic regulating factors in neural stem cell proliferation/differentiation balance.

The role of p75NTR in neurogenesis and neural stem cell niches is debated with both positive [189] and negative results [190]. Young et. al. have recently shown that a small population of cells within the stem cell niche of the rat subventricular zone (SVZ) expresses p75 NTR and that these cells are responsible for neuron production in both newborn and adult animals. On the other hand Bath *et al* observed no effect on cell proliferation or survival in SVZ of p75NTR null mice. 

#### Epigenetic Factors

Chromatin modifications that are not necessarily heritable but still result in changes of gene expression can be defined as epigenetic factors. Processes that can modulate DNA or associated structures independently of the DNA sequence, such as DNA methylation, histone modification, chromatin remodelling and transcriptional feedback loops, are thought to constitute the main epigenetic mechanisms. Importantly, although epigenetic effects are relatively long-lasting, it is the change in epigenetic programs that helps to choreograph the precisely timed transitions from one cellular state to another in coordination with both internal and external cues during adult neurogenesis.

Epigenetic modifications can be broadly divided into three major types: DNA methylation of CpG dinucleotides, covalent modifications of histone tails, and pre- and post-transcriptional modifications elicited by small non-coding RNAs (ncRNAs) [191]. Besides the direct effect of these modifications on gene transcription, epigenetic modifications can also act as platforms for chromatin-remodelling complexes, leading to more permanent changes in the chromatin state that can result in longer-term gene activation/silencing. Collectively, these changes result in the stabilization of transcriptional programs ultimately affecting the cellular phenotype.

##### Epigenetic Maintenance of the Neural Stem Cells

The polycomb group protein Bmi1 is a key epigenetic regulator of the self-renewal property and of maintenance of neural stem cells [192-195]. Bmi1 is a member of the PcG complex that catalyzes H3K27 methylation; in Bmi1 knockout mice, SVZ-derived adult NSCs (but not lineage-restricted progenitors) are depleted [194]. The methyl-CpG binding protein 1 (Mbd1] has emerged as a crucial and specific regulator of adult neural stem cells in the SGZ. Mbd1 knockout mice show no detectable developmental defects and appear healthy throughout life, but they have severely reduced adult neurogenesis and impaired spatial learning. Mbd1 binds to the promoter of the gene that encodes FGF2, a mitogen for adult neural progenitors, and regulates its expression in adult SGZ neural progenitors in a manner that depends on DNA methylation [196, 197]. The regulated expression of FGF2 in adult NSCs might allow temporally appropriate neuronal differentiation both *in vitro *and *in vivo*. Surprisingly, as a methyl-CpG ‘reader’ protein, Mbd1 also seems to affect DNA methylation levels *per se*, suggesting that Mbd1 may recruit unidentified DNA methyltransferases to form propagating feedback loops that silence its target genes over many rounds of cell division. Such long-term silencing propagation would, in principle, be similar to the action of the PcG complex in promoting the self-renewal of adult neural stem cells in the SVZ, although these two mechanisms enact different epigenetic silencing machineries in different brain regions [198]. 

##### Epigenetic Regulation of Neural Stem Cell Differentiation

Unlike the silencing Polycomb Group PcG (PgC) complex, the Trithorax Group (TrxG) proteins establish stable and transcriptionally active chromatin domains by catalyzing and maintaining histone tail H3K4 methylation [199]. One TrxG member, Mll1 (mixed-lineage leukemia 1], encodes an H3K4 methyltransferase. Mll1 is specifically required for neuronal, but not glial, differentiation from adult neural stem cells [200]. The homeobox protein Dlx2 has been identified as one direct target of Mll1 and is crucial for neurogenesis in the SVZ.

Inactivation of the histone deacetylase HDAC activities by HDAC inhibitors leads to marked enhancement of neuronal differentiation from adult neural stem cells in the SGZ [201]. In these stem cells, HDAC silences the expression of key neurogenic transcription factors, such as NeuroD1, and of cell-cycle regulators through gene-specific recruitment by the transcription factor Tlx1 [202]. A deficiency of one member of the HDAC family, HDAC2, results in specific and cell-autonomous defects in neural differentiation during adult but not embryonic neurogenesis [203]. Many members of the small RNA family that regulate adult neurogenesis have been identified. miR-184 functions as a direct target of Mbd1 to inhibit neuronal differentiation from adult neural stem cells in the SGZ [204] by post-transcriptional repression of Numb-like, a regulator of neuronal differentiation during development. miR-137 has been identified as a direct target of Sox2 and of another DNA methyl-CpG-binding protein, MeCP2, which inhibits neuronal differentiation and maturation in adult SGZ neural stem cells [205]. One of the most abundant microRNA in the adult brain is miR-124, which is both required and sufficient to promote neuronal differentiation from adult SVZ neural stem cells [206]. Interestingly, one crucial target of miR-124 action in this system is another Sox family protein, Sox9. Functionally, miR-124-mediated repression of Sox9 ensures correct cell state progression along the SVZ stem cell lineage to neurons [206].

In addition, small modulatory RNA has also been shown to trigger neuronal gene expression from adult neural stem cells by inhibiting the action of the REST-NRSF (Repressor element-1 Silencing Transcription Factor and Neuron Restrictive Silencer Factor) transcriptional machineries [207]. REST-NRSF potently represses neuronal genes, partly by suppressing miR-124 and switching the ATP-dependent chromatin remodelling complexes during neural differentiation [208, 209].

For detailed review of epigenetic control of NSC differentiation see ref. 198, 208 and 210.

### Extrinsic Factors: the Niche Microenvironment

Stem cell niche is defined as a special portion of tissue capable of hosting and maintaining the stem cells for the lifetime. It ensures a unique microenvironment where interactions between stem cells and resident niche cells or extracellular matrix molecules and soluble autocrine, paracrine and even endocrine signals [211], provide the proper control of the stem cell properties. The niches may modify their signals in response to changing conditions to ensure that stem cell activity meets the needs of the tissue. 

A special role in homeostasis and function of NSC niches is played by the vasculature and CSF. Indeed, specialized neurovascular interfaces, which support adult NSC functions, have been described [22, 32]. SVZ blood vessels appear to lack astrocytes end-feet and pericyte coverage. These gaps may provide enhanced access to different vascular components of the niche (possible sites of diffusion of circulating factors). Moreover, endothelial cell-derived factors have been shown to stimulate the proliferation and differentiation of NSCs during development and in adulthood [33, 212]. Furthermore, ECM-basal lamina associated to the vessels also exhibited trophic properties on NSCs [69]. 

Another important interface of the NSC niche is the cerebral spinal fluid [213]. Important signals generated in distant location may diffuse to the niche by CSF to modulate the NSC function and proliferation. 

In the last years, many features of the NSC niche microenvironment have been revealed, including cell types, soluble factors and ECM components. On the other hand, a comprehensive characterization is still lacking.

#### Chemoattraction to the Home 

Cells of the NSC pools have different stationary or migratory properties depending on their stage of maturation and on the signals converging in the niche. Quiescent stem cells are kept safely inside the niche, while neural precursors born in the SVZ may cross long distance anteriorly through the rostral migratory stream and reach the olfactory bulb (OB), where they differentiate into inter-neurons. In the SGZ neuroblast are generated locally and subsequently migrate for short distance to integrate in the dentate gyrus [214]. Major molecular mechanisms involved in homing and migration of NSCs are described. 

##### Stromal Cell-derived Factor-1 

The chemokine stromal cell-derived factor-1 (SDF-1, also referred to as CXCL12) regulates many relevant biological processes, including neuronal development, stem cell motility, neovascularization and tumorigenesis [215]. SDF-1 signals *via* its receptors, the CXC chemokine receptor 4 (CXCR4], and provides migratory cues to hematopoietic stem cells and leukocytes. Recently the novel CXCR7/RDC1 receptor has been identified as another SDF-1 ligand [216-218]. The interaction between SDF-1 and CXCR4 can be blocked by the antagonist AMD31000 [219]. 

Both SDF-1 and CXCR4 are constitutively expressed in the developing and adult CNS. In early developmental stages, expression of CXCR4 is mainly detected in the ventricular zone, SVZ and marginal zone, which are specialized niches for survival and proliferation of neural precursors. In adult CNS, SDF-1/CXCR4 expression persists within the main NSC niches, i.e. SVZ and SGZ, as well as the central canal and the meninges.

Major cell sources of SDF-1 in adult brain are endothelial and meningeal cells [220, 221]. SDF1/CXCR4 signalling has been implicated in CNS cell migration. In the developing brain cortex, Cajal-Retzius cells are a transient population of neurons located in the marginal zone, directly adjacent to the meninges [222]. Expression of CXCR4 is detected in these cells from preplate (E13.5) to early postnatal stage (P3] [150] whereas expression of SDF-1 is detected also in the meninges [150, 223]. In mice knock-out for either the ligand or its receptor, the Cajal-Retzius appear dispersed and displaced into deeper cortical layers [223, 224], suggesting that CXCL12/CXCR4 may influence the early localization of these cells. 

Regulated expression of SDF1 in the intermediate SVZ influences lateromedial tangential migration of CXCR4-expressing GABAergic neurons [160]. Moreover, SDF-1 dependent chemotaxis has been described in SGZ [225]. SDF1/CXCR4 signalling also directs the migration of sensory neuron progenitors to the dorsal root ganglia (DRG) in mice [226]. 

CXCR4 is expressed on all stages of the SVZ lineage, but SDF1 has differential effects on the progenitor stages. Recently, ependymal and endothelial cells have been reported to be able to produce a U-shaped gradient of secreted SDF-1. High levels of SDF1 from ependymal cells stimulate quiescence of NSCs. In a subset of activated NSCs expressing EGFR, this factor stimulates movement toward the blood vessel surface, proliferation, and generation of transient amplifying cells [227].

*In vitro* SDF1 increases proliferation of cultured rat or human NSCs in a concentration-dependent manner [228, 229]; on the other hand other authors have reported that SDF1 is devoid of proliferating activity but increases differentiation of adult mouse NSCs [230] or maintains human foetal NPCs in a quiescent state [231]. Overexpression of CXCR4 decreases proliferation of adult rat SVZ NSCs; however, this effect was abolished when SDF1 was added at high concentrations [229], suggesting that the balance between availability of SDF1 and receptor level are important regulators of NSC proliferation.

SDF-1 is also implicated in oligodendroglia ontogeny both *in vitro* [232] and *in vivo* [233].

#### Oxygen Tension 

Quiescence, proliferation and cell-fate can be influenced by oxygen tension in embryonic, hematopoietic, mesenchymal, and neural stem cells [234, 235]. The oxygen partial pressure of inspired air (pO_2_= 21%, 160 mm Hg) progressively decreases and has dropped to 2%-9% [14-65 mm Hg) in peripheral tissues [236]. In the human brain, pO_2_ measured using partial pressure catheter electrodes varies from approximately 3% (23.2 mm Hg) to 4% [33 mm Hg) [237]. 

The cellular molecular responses to changes in oxygen tension include the endoplasmic reticulum (ER) stress response and activation of hypoxia-inducible transcriptions factors (HIFs), oxygen sensitive ion channels and the environmental sensing mammalian target of rapamycin (mTOR) [238, 239]. Low oxygen tensions (hypoxia) (in the range of 1%-9%), is a condition where the risk of oxidative stress by generation of reactive oxygen species decreases [240]. Hypoxia has been shown to regulate Oct4 and Notch signalling [241]. 

Oxygen tensions influence NSC stemness and fate by modulating intracellular pathways including p53 and Notch signalling [242, 243]. Hypoxia also enhances *in vitro* proliferation of NSCs by activation of the JNK signalling [244].

#### Autocrine, Paracrine and Endocrine Soluble Factors

##### Neurotrophic Factors

Nerve growth factor (NGF), brain-derived neurotrophic factor (BDNF), neurotrophin-3 (NT-3] and neurotrophin-4/5 (NT-4/5] are essential factors in the developing CNS. Neurotrophins are synthesized as precursor forms (proneurotrophins). Mature forms of neurotrophins exert their effects by binding to specific tyrosine kinases receptors (TrkA, TrkB and TrkC) and to the p75 receptor, while proneurotrophins interact with the receptor p75.

Brain- derived neurotrophic factor (BDNF). 

BDNF mediates its diverse actions by binding to the TrkB and the p75 receptors. The two forms of BDNF (pro- and mature BDNF) have been shown to play divergent roles during neuronal development and in adulthood [245-249]. 

BDNF promotes the survival and differentiation of a variety of neuronal populations [245]. Infusion of BDNF into the lateral ventricles of adult rats causes a large increase of newborn neurons in the olfactory bulb [246], in the striatum and septum [247]. Disruption of BDNF signalling leads to impairments of cortical development [248]. 

Indication that BDNF is an important niche factor able to promote neurogenesis comes from studies showing that its ectopic overexpression in non-neurogenic regions, such as the striatum, can support the survival of grafted progenitor cells [249]. 

###### Neural Growth Factor (NGF)

NGF promotes survival, neurite growth and neurotransmitter production of cholinergic neurons of the basal forebrain system, of nociceptive dorsal root ganglion neurons and of some third-order sympathetic neuron [250, 251]. Intraventricular administration of NGF can increase SVZ proliferation *in vivo* [252]. Interestingly, chronic intracerebroventricular infusion of NGF induces hyperplastic changes of the leptomeninges of the rat and the monkey [253]. This proliferative response was limited to the leptomeninges without evidence for participation of the brain or spinal cord parenchyma, and without changes indicative of neoplastic transformation. *In vitro* treatment of neurospheres with NGF resulted in differentiation of bipolar neuronal cells and of cholinergic neuronal cells expressing choline acetyl-transfer and tyrosine hydroxylase [254]. NGF has also been shown to natively bind to collagen and laminin [255]. In addition, some of the effects of NGF are also dependent on the presence of other factors. For example, the mitogenic response of neuronal precursor cells to NGF in cultures of striatal primordium requires previous exposure to FGF [256].

###### Ciliary Neurotrophic Factor (CNTF)

CNTF is a growth factor that is exclusively expressed within the CNS [257]. In the adult SVZ, exogenous CNTF activates the Notch signalling pathway [258] by interacting with its receptor CNTFR-alpha expressed by a subset of GFAP positive cells [259]. Inhibition of CTNF activity by antibodies or gene knock-out is associated to reduction in cell proliferation within the SVZ [258, 260]. Some of the neurotrophic effects of dopaminergic innervations are related to CNTF release, thus providing a mechanistic explanation for reduced SVZ cell proliferation in Parkinson Disease [260]. CNTF is also a potent regulator of adult neurogenesis in brain regions other than the SVZ, including the dentate gyrus and the hypothalamus [63, 66, 258, 261]. 

*In vitro* CNTF inhibits glial cell fate restriction in uncommitted neurosphere cells, resulting in the maintenance of the NSC phenotype, while accelerating the differentiation of progenitors already committed to the astrocytic lineage [262]. CTNF, together with other molecules including LIF and IL-6, promotes the survival of mature oligodendrocytes in culture [263]. 

##### Other Trophic Factors

###### Bone morphogenetic proteins (BMPs)

BMPs are 20 different growth factors interacting with receptors of the BMPR family. During development, several BMPs, including BMP4, are involved in repression of the oligodendroglial lineage and generation of the astroglial lineage [264]. In the adult SVZ, BMPs (BMP2, BMP4, BMP7] direct astroglial differentiation and inhibit neurogenesis, whereas the secreted polypeptide Noggin inhibits the action of BMP4 in the SVZ and favours differentiation of neurons over glial cells [265]. Noggin was also shown to induce neuronal differentiation of NSCs when transplanted into the neighbouring areas of the brain [266], suggesting that BMP4 is critical for neuronal differentiation of stem cells of the SVZ. 

Downstream targets in the BMP pathway, such as Smad4, are also critical for normal neurogenesis. A recent study has shown that Smad4 is expressed exclusively in progenitors of the SVZ and not in the SGZ. Deletion of Smad4 in SVZ GLAST+ stem cells decreased the number of neuroblasts generated and increased the number of Olig2+ oligodendrocyte precursors that migrated to the corpus callosum. Inducible deletion of Smad4 from either stem cells showed that Smad4 signalling was only required at the earliest stage of SVZ neurogenesis for proper neuronal development [267]. In general, available data suggest that BMP signalling is essential also in adult neurogenesis.

###### WNT

The Wnts are a family of secreted signalling glycoproteins that regulate cell proliferation, fate decision and differentiation. WNT is an important signalling molecule in CNS development as indicated by its role in mesencephalon and cerebellum induction. [268]. Moreover, Wnt-1, Wnt-3a, and Wnt-5a play a role in dopaminergic neuron development [269] and Wnt signalling regulates adult hippocampal neurogenesis [269]. Lie *et al*. have shown that blockade of Wnt signalling suppressed, while stimulation of Wnt signalling enhanced, SGZ neurogenesis [270]. 

###### Other Growth factors

EGF and FGF-2 are two growth factors important for the maintenance and proliferation of NSCs. Exogenous EGF can induce the differentiation of adult NSCs into glial cells *in vivo* [271] whereas FGF-2 stimulates NSC proliferation [272]. 

Insulin-like growth factor-1 (IGF-1] is a growth-promoting peptide that is important during development of the brain [273]. In IGF-1 KO mice, the number of granule cells in the hippocampus, the density of oligodendrocytes and neurons within the olfactory bulbs, and total brain size are reduced [274]; on the contrary, overexpression of IGF-1 results in an increase in brain size and myelin content [275]. IGF-1 receptors are expressed in the adult dentate gyrus [276] and can be activated by either endocrine [277] or paracrine mechanisms [278]. Peripheral infusion of IGF-1 increases proliferation of SGZ progenitor cell and selectively induces neurogenesis in the progeny of adult neural progenitor cells [279]. 

Leukemia Inhibitory Factor (LIF) has been shown to support NSC self-renewal [280] and promote the survival of mature oligodendrocytes *in vitro* [263]

Neurosteroids, such as dehydroepiandrosterone (DHEA), pregnenolone and their sulphate esters, progesterone and allopregnanolone are synthesized in CNS by neurons and glia in concentrations high enough to exert paracrine effects [281]. Neurosteroids affect neuronal survival, neurite outgrowth and neurogenesis [282]. Along with their neuroprotective and prosurvival effects, neurosteroids appear to affect neurogenesis. DHEA uniquely increases the number of newly formed neurons in the rat dentate gyrus of the hippocampus. Interestingly, DHEA also antagonizes the suppressive effect of corticosterone on both neurogenesis and neuronal precursor proliferation [283].

Other soluble factors are produced by endothelial cells, which are a cellular component of the NSC niche. This is the case of VEGF that has been shown to positively regulate neurogenesis [284]. Astrocytes also appear to be a source of locally acting trophic factors for NSCs [285].

##### Endocrine Signals 

High prolactin levels during pregnancy promote adult neurogenesis in the subventricular zone of the lateral ventricle (SVZ) of the maternal brain of mice and rats [286]. Newly generated SVZ neurons migrate *via* the rostral migratory stream and are incorporated into the olfactory bulb where they functionally contribute to olfactory learning of novel odorants [287]. Although some studies observed a negligible response to prolactin within the DG in untreated healthy animals, others have shown that prolactin protects neurogenesis in the DG of chronically stressed mice and promotes neuronal fate [288]

Erythropoietin (EPO) synthesis can be activated in astrocytes and neurons, but circulating EPO may also reach NSC niches by crossing the blood-brain barrier. Epo/EpoR signalling promotes cell survival in embryonic brain and contributes to neural cell proliferation in adult brain in regions associated with neurogenesis [289]. In a mouse model of ischemia, EPO acts as paracrine neuroprotective mediator [290] and promotes the migration of neuroblasts by inducing the secretion of the matrix metalloproteinase-2 and -9 [291]. Administration of recombinant human EPO in an animal model of spinal cord compression was associated to early recovery of function as well as to neuroprotective, anti-inflammatory and antiapoptotic effects [292]. Interestingly, exogenous EPO can affect NSC differentiation into neurons [293].

##### Inflammatory Cytokines in NSC Niche in “Resting” Condition

Cytokines known to have pivotal roles in immune reaction have also been shown to influence synaptic plasticity, neurogenesis and neuronal survival [294-296]. Some of these effects occur in absence of inflammation as in the case of IL-1β that plays a role in NSC fate specification and participates in the regulation of neuronal proliferation during spinal cord development [297]. Similarly, IL-1α increases the expression of genes associated with neurogenesis during neuronal induction. This has been demonstrated to have functional significance since transplantation of transgenic neural precursor cells overexpressing IL-1ra improves chronic isolation-induced impairment of memory and neurogenesis [298]. The effects of some cytokines vary depending on concentration. For example, physiological concentrations of IL-1β increase the differentiation of neural progenitors to dopaminergic neurons *in vitro* in both rodents and humans [299]; on the contrary, chronic high (inflammatory) concentrations of IL-1β inhibit neurogenesis [300, 301], possibly by NF-κB signalling [302]. Similarly, interleukin-6 (IL-6] promotes differentiation of NSC to neuronal lineages through the Janus kinase (JAK)/STAT pathway at relatively low concentrations [303] and promotes survival of differentiated neurons, astrocytes and oligodendrocytes [304, 305]. By contrast, exposure to high levels of recombinant IL-6 (50 ng/ml) or TNF-α (20 ng/ml) decreased *in vitro* neurogenesis by approximately 50% [306]. 

Action of TNF-α in brain is complex and depends on the target cells and receptors engaged. Evidence from TNF-α receptor knock-out mouse suggests that signalling through TNF-R1 suppresses neural progenitor proliferation and neurogenesis *in vivo*, whilst signalling through TNF-R2 enhances neurogenesis in basal conditions or in neurodegenerative disorders [307]. Under pathological conditions inhibition of proper TNF receptor trimerization by the TNF-α converting enzyme TACE/ADAM17 induces neural progenitor proliferation in the SVZ [308]. 

As most of the inflammatory cytokines, also IFN-γ displays dual effects on neurogenesis. It inhibits NSC proliferation and reduces NSC survival in a dose-dependent manner and synergistically with TNF-α [309]. On the other hand, it promotes neural migration, differentiation and neurite outgrowth of murine adult NSCs [310] *via* c-Jun N-terminal kinase (JNK) pathways [311]. 

Several other chemokines and cytokines have been identified as important regulators of NSC functions. Among them IL-4 has been reported to induce neurogenesis and oligodendrogenesis from adult NSCs [312]. Leptin inhibits differentiation of multipotent and/or glial progenitor cells into oligodendrocytes precursors in the mouse embryonic cerebral cortex [313]. The monocyte chemotactic protein 1 MCP-1 acts as chemotactic factor of neural precursor [314] while the chemokine (C-C motif) ligand 2 CCL2 promotes neuronal differentiation of SVZ neural progenitors [315].

###### Resident Niche Cells 

In the niche NSCs and resident cells share close anatomical relationship allowing many cell-to-cell interactions and paracrine stimulations. For example, in hippocampus mature astrocytes promote neurogenesis, whereas neurons increase oligogenesis by modulating NSC differentiation. Here we provide a brief summary of the main niche cell populations and of their contribution to NSC homeostasis. 

##### Astrocytes 

Although during development most of the parenchymal astrocytes are generated after neurons, they regulate synapse formation and synaptic transmission and are capable of controlling neurogenesis by instructing NSCs to adopt a neuronal fate [316]. In SGZ and adult hippocampus protoplasmic astrocytes release FGF2 which stimulates proliferation of FGFr1-expressing NSCs. Astrocytes also secrete other factors that induce neuronal differentiation of NSCs, including NFG and BDNF [303]. Parenchymal astrocytes do not divide in the healthy brain. When cultured *in vitro*, adult parenchymal astrocytes do not form self-renewing and multipotent neurospheres. However, upon injury they become hypertrophic, change their morphology and upregulate the expression of GFAP, vimentin and nestin and start to proliferate; these reactive astrocytes show NSC features when cultured *in vitro *[317].

##### NG2-Oligodendrocyte Precursor Cells (NG2-OPCs)

Parenchymal NG2-OPCs should not be confused with NG2 pericytes which have a perivascular location. Beyond the NSC potential described on the section dedicated to the cortical niches, NG2-OPCs have been shown to interact with other neural cells located along the border of the SVZ and the rostral migratory stream. The abundance of NG2 cells increased in the distal parts of the rostral migratory stream and especially in the olfactory bulb, where NG2 cell processes are in close proximity to many maturing interneurons [318].

NG2-OPCs express voltage-dependent ion channels and neurotransmitter receptors [319] and form neuron-polydendrocyte synapses [320]. Although depolarizing spikes were detected in polydendrocytes, their functional significance is still unknown [321]. NG2 cells provide an adhesive substrate for axonal growth cones and promote their growth both *in vivo* and *in vitro* suggesting that these cells facilitate axonal growth during development and regeneration. [322]

##### Immune Cells 

For long time it has been thought that CNS is an ‘immune privileged’ organ. Indeed the brain parenchyma does not contain lymphatic vessels and is devoid of classical antigen-presenting cells (APCs), such as dendritic cells [323]. CNS is protected from invading microorganisms by the endothelial blood-brain barrier (BBB), the epithelial blood-cerebrospinal fluid barrier and the innate immune cell population, the resident microglia.

Microglia is the main immune effector cell in the nervous system and it serves as scavenger for damaged neurons, plaques, and infectious agents [324]. Microglia populates the CNS throughout adult age; it remains in a quiescent state, and rapidly proliferates only upon activation [325]. As immune effector, microglia has chemotactic properties modulated by endogenous and exogenous chemotactic factors, including SDF-1 gradient. 

In the main neurogenic niches (SVZ and SGZ) microglia is in close contact with NSCs and *in vivo *evidence suggests that it regulates neurogenesis [326, 327]. *In vitro* experiments (coculturing or microglia-conditioned medium) indicate that these cells provide diffusible factor(s) essential for neurogenesis, but not for NSC maintenance and self-renewal [328]. In healthy physiological conditions, almost no immune cells infiltrate the brain parenchyma from the circulation. A continuous leukocyte trafficking in healthy CNS only occurs in restricted areas such as the CSF/CNS borders. Choroid plexus and meninges contain APCs that are located in the choroid plexus stroma outside the barrier (choroid plexus macrophages), in sub arachnoid space (meningeal macrophages), and on the apical surface of choroid plexus epithelium (Kolmer cells) [329]. Interestingly, these resident macrophages can secrete several trophic factors including IGF-1, BDNF and NGF [330]. In general leukocytes penetrate the CNS parenchyma in the case of an inflammatory process. However, over the past decades a new function has emerged as circulating leucocytes have been shown to have an essential function in supporting brain plasticity [331]. In particular, impaired neurogenesis has been observed in both SVZ and SGZ of T-cell-deficient mice [i.e. severe combined immune deficiency (SCID) and nude] compared to that in wild type mice [327]. Moreover reconstitution of the T-cell pool in immune-deficient mice by injection of wild-type splenocytes showed a partial restoration of proliferation and neuronal differentiation that reached levels similar to those observed in wild-type mice [327, 332] Experiments in models of CNS injury have shown that CNS autoreactive T cells exert neuroprotective effect [333-335]. It is remarkable to note that, blood born leucocytes were found to support the formation of new spinal cord [145, 336] and retina neural progenitors [337]. Consistent with this observation we have found a significant decrease (>3 fold, p<0.0001) of nestin-positive cells in meninges of the parietal cortex of adult SCID mice (unpublished observation).

The mechanism by which leucocytes influence adult neurogenic niches seems to involve T cells within the choroid plexus and meninges that may interact locally with antigen-presenting cells (such as dendritic cells and macrophages) and secrete cytokines or growth factors into the cerebrospinal fluid [338]. 

This mechanism appears to have functional significance, since CNS-specific T cells are required for spatial learning and memory and for the expression of BDNF in the dentate gyrus [311]. The cross-talk between the brain and the immune cells has been shown to be required to positively support several forms of CNS plasticity [328]

Additionally NSCs have been found to express Toll-like receptors (TLRs), molecules usually associated with innate immunity in mammals. In particular, TLR-2 signaling positively regulates neuronal differentiation, whereas TLR-4 signaling negatively regulates NSC proliferation [339, 340]

#### Brain Vasculature, Blood-Brain-Barrier and Cerebrospinal Fluid 

Brain interfaces play an essential role in CNS homeostasis. At the level of the blood-brain and blood-CSF barriers, the interactions between astrocytic endfeet, mural cells (vascular smooth muscle cells and pericytes) and endothelial cells are crucial for the delivery of essential metabolic substances and for restricting the diffusion of dangerous agents from the blood to the neural tissue. 

Brain blood vessels are highly heterogeneous. There are the large arteries of the circle of Willis that branch out into smaller pia arteries and arterioles. Pia arteries travel along the surface of the brain across the subarachnoid space and penetrate in the parenchyma. The perivascular space surrounding vessels as they enter the brain (Virchow-Robin space) is formed by the extroflexions of leptomeninges (arachnoid and pia mater) filled with CSF [341]. Progression to capillaries yields the disappearance of the Virchow-Robin space, allowing astrocytic end-feet to directly contact vascular cells and their basement membrane. In the brain capillaries the endothelial cells are surrounded by pericytes or by the pericyte processes and the extracellular matrix. 

At the level of the NSC niche, the vasculature plays a fundamental role. Indeed, a specialized neurovascular interface supporting adult NSC functions has been described [26, 32]. SVZ blood vessels lack astrocyte end-feet and pericyte coverage. These gaps may provide enhanced access to different vascular components of the niche as well as allow diffusion of circulating factors relevant for the niche homeostasis and the identity of NSCs [32, 33, 212]. 

The tight connection between vessels and NSCs is shown by the observation that endothelial cell-derived factors stimulate the proliferation and differentiation of NSCs during development and in adulthood [33, 212]. Indeed, several vascular-derived molecules locally regulate the adult NSC niche including autocrine/paracrine factors, e.g. LIF, BDNF, VEGF [284], PDGF, [342] and laminins. Other vessel-derived factors that influence NSC biology are the growth factors FGF-2, EGF, the stem cell factor (SCF), IGF-1, the cytokines TGF-β and IL-6, BMPs, SDF-1, collagen IV, Eph/ephrins, angiopoietin, and erythropoietin [343]. In addition the ECM-basal lamina associated to the vessels also exhibits trophic properties for the NSCs [69]. In SVZ, NSCs extend a long basal process terminating on the blood vessels in the form of specialized endfeet that may sense changes in the perivascular ECM [25, 32].

Pericytes regulate BBB-specific gene expression patterns in endothelial cells and induce polarization of the astrocyte end-feet surrounding CNS blood vessels [344]. Brain capillaries pericytes express nestin, NG2, smooth muscle α-actin, desmin, PDGFR-β, aminopeptidase A and N; none of these markers is specific or expressed by all the pericyte sub-sets [345]. The neural stem cell-like potential of NG2/nestin pericytes has been described [346]. Recently, genetically labeled perivascular cells have been shown to be the major component necessary to the scar formation after spinal cord dorsal funiculus incision or dorsal hemisection [347]

The cerebrospinal fluid (CSF) is secreted by the choroid plexuses located in the lateral, third and fourth ventricles and meninges. In CSF the restricted diffusion at the blood-choroid plexus barrier and basolateral membrane hinders diffusion from plasma to CSF of hydrophilic solutes larger than 60 kDa [348]. However, molecules produced over the barrier, can flow through all the ventricular regions and through the meningeal perivascular space and reach a very large portion of the CNS. Indeed, CSF contains growth factors and other neurotrophic factors, which are important for cell survival and proliferation. The CSF may deliver secreted proteins to several regions, including FGF-2 to midbrain progenitors [349], Sonic hedgehog to cerebellar progenitors [350] and guidance of neuroblasts in adult brain [351]. Recently, CSF has been demonstrated to modulate in an age-dependent manner NSC proliferation, mainly, but not exclusively, *via* IGF-2 [213]. 

#### Extracellular Matrix Interactions

The molecules of the highly organized extracellular matrix (ECM) play important roles in the development of the CNS. The ECM fills out all the extracellular space in the adult CNS and accounts for approximately 20% of the total volume of the mature brain [352]. A basal lamina-like ECM is present at the blood-brain barrier and in neurogenic niches. A highly organized ECM-rich basal lamina within the NSC niche provides both structural support in orienting the cells and instructive information for cell homeostasis. The basal lamina/basement membranes are thin sheets of ECM that are composed of collagen IV, nidogen, perlecan, agrin and members of the laminin family [353]. Laminins are recognized by at least three major classes of cell surface receptors: integrins, syndecans, and dystroglycan [354]. Integrin receptors, such as α6β1 (one of the receptors for laminin), are present on the surface of stem cells, boost the ability of the niche to connect NSCs into a functional network and may directly affect their proliferation [26]. In addition to provide a physical contact site, components of the ECM, such as N-sulphate heparan sulphate proteoglycans (HSPG) bind FGF-2 thereby concentrating this growth factor in the niche (69]. Additional effects of ECM factors include the formation of highly dynamic filopodia-like processes on axons and dendrites induced by the transmembrane form of the extracellular matrix heparan sulfate proteoglycan agrin [355]. NGF has been shown to bind to collagen [255] and laminin [356] a mechanism whereby this neurotrofic factors can be concentrated and persists at the level of the niche.

#### Other NSC Niche Signals

##### Activity-Dependent Influence of the Niche

Various cell types in the brain have been shown to play interesting roles in extrinsic regulation of neurogenesis; among these, mature neurons play an important role mostly mediated by activity-dependent events.

Neuronal activity modulates neurogenesis at different levels, from proliferation of adult neural progenitors, to differentiation, maturation, integration and survival of newborn neurons in the adult brain [357]. The effects are in part dependent on release of classical neurotransmitter and activation of their receptors.

GABA, the major neurotransmitter used by mature interneurons in the brain, causes depolarization of neural progenitors and immature neurons in the adult brain because Cl^-^ has an outward electrochemical gradient [358]. GABA is released from migrating neuroblasts and local interneurons in the SVZ where it regulates NSC proliferation [359]**.** In the SGZ, both tonic and phasic GABAA receptor activation induce neuronal maturation and synaptic integration of newborn granule cells [360].

Ionotropic glutamate receptors are also present in immature neurons and early progenitors [361]. Activation of the glutamate ionotropic NMDA receptor promotes the proliferation of adult hippocampus neural progenitors and the survival of newborn neurons in adult hippocampus [361]. 

Stimulation of dopamine receptors leads to significant increases in SVZ neurogenesis [362-364]. The effects of dopaminergic transmission are at least in part dependent on subtypes of dopamine receptor that are activated [364]. Ablation of dopaminergic neurons of the substantia nigra decreased NSC proliferation in the SVZ [365].

##### Exosomes/Microvesicles 

Exosomes/microvesicles were ﬁrst found as a mechanism for shedding membrane proteins such as transferrin receptors during the maturation of reticulocytes, it is now believed that they are involved in a wide range of physiological functions such as intercellular communication, membrane exchange between cells, and as an alternative to lysosomal degradation [366]. Human NSCs have been shown to secrete exosomes [367]. This 20-100 nm particles could be a novel mechanism by which proteins, microRNA, and even DNA can be exchanged between NSCs and resident niche cells. Future studies will shed light on this topic and their role in NSC biology.

#### Remarks on NSC Location and Control by Intrinsic and Trophic Factors

The analysis of distribution of *bona fide* NSCs indicates that niches are present in a number of regions of the CNS and that types and combination of intrinsic and/or extrinsic factors vary in different locations. Thus extrinsic signals may influence the plasticity of the neural precursors as much as the epigenetic state of the precursors may affect the responses to environmental stimuli. 

These remarks raise the question of the origin of the NSCs in the different location. The idea of the SVZ acting like a beating heart providing the different niches of a continuous flux of NSCs requires the existence of signals controlling NSC migratory activity. Indeed, factors involved in cell migration and/or homing are part of the external signals acting in niches. 

In addition, pathways for facilitated flux of cells can be postulated. In this context we want to point out the tight connection existing between a niche on one side and the vasculature and the CSF on the other. This connection should be considered not only as pathways for delivery of activation signal(s) to a quiescent niche cell population; it could also be considered as a facilitated path for precursor cell delivery to the site of integration in the tissue network. Meninges, which envelop the vasculature, can thus serve both as niche as well as tool for activated NSC/precursor delivery from one niche to the other and, locally, from a niche to the site of integration in the tissue. In line with this possible function of meninges as transient home for migrating NSCs are our preliminary observation that NSC injected into the ventricle acquire a meningeal location [Fumagalli G., Decimo I., Bifari F., Krampera M. Homing and migration of transplanted Leptomeningeal Stem/progenitor Cells in adult rat brain. Program No. 34.12/E16. 2010 Neuroscience Meeting Planner. San Diego, CA: Society for Neuroscience, 2010. Online]. Insights on the mechanisms of NSC activation and on the possible connection between niches should be obtained by the analysis of physiological and pathological events associated to NSC activation. To this aim, the literature on these aspects of NSC biology will be reviewed in the following two sections.

## FUNCTIONAL SIGNIFICANCE OF ADULT NEURAL STEM CELL NICHES 

How much plastic is the brain and which are the strategies that the brain uses to face continuously changing clues and maintain its homeostasis?

The brain is a complex structure formed by different cell types (both neural and non neural) that all together coordinately contribute to the adaptation to the external environment. Physical activity, enriched environment, aging, stress, and learning are external stimuli that induce adaptive plastic responses that involve neurogenesis. Thus, the generation of new neurons - *plasticity by addition* - is a possible strategy to generate plastic responses to the external environment that adds to the well known synaptic-mediated plasticity events.

The first approach for studying neurogenesis is identification of proliferating cells in the brain. Vellema *et al* constructed a detailed quantitative 3D map of proliferating cells in adult brain of songbird [368]. Proliferating cells (detected after 24h of challenge with BrdU) were present in the lateral ventricle and, to a much lesser extent, in the brain parenchyma, including midbrain, hindbrain, and cerebellar structures. They also observed cells stained by BrdU 38 days after loading. In this case 93% of the BrdU-positive cells in the telencephalon were positive for the early neuronal phenotype marker Hu. A similar map has not been drawn for mammals. Although, it is general consensus that under physiological conditions, adult neurogenesis is restricted to the olfactory bulb and dentate gyrus of the hippocampus, there are no accurate indication on fate, distribution and migratory activity of the proliferating cells. This information is relevant and may help to elucidate the significance of the adult neurogenesis.

At the moment little is known on the functionality of the newly generated neurons. In the majority of the studies on neurogenesis most of the new neuronal cells express markers of immature neurons (i.e. doublecortin). On the other hand, the presence of this marker should not be considered indication or prediction of functional integration of new neurons in the tissue. This aspect should be studied by combining cell labeling strategies (transgenic animals or retroviral transfection) and electrophysiological recording [369]. For a review on the current viral tracing methods, heterologous receptor expression systems and optogenetic technologies that can be used to clarify the integration into the neuronal circuits of adult-born neurons see Arenkiel *et al*. [370]. Indeed, the combination of morphological and electrophysiological studies has demonstrated that adult-born SGZ neurons display all the characteristics of mature neurons within the hippocampus network [369].

In the following pages we will review some of the experimental models used for studying NSC contribution to physiological functions.

### Models for Physiological Modulation of Neurogenesis

#### Enriched Environment and Physical Activity

A well established model for studying adult neurogenesis is the “enriched environment” [371]. With this model, mice showed a consistent increase in the number of new hippocampus granule cells [372-374]; the acute effect was due to enhanced survival on the progeny of dividing precursor cells and not to increased divisions of the precursor cells [375]. Over longer periods of exposition to the enriched environment the number of precursor cells also increased as result of increased proliferation rate [376, 377].

Other information was provided by models of partial hippocampus ablation. In a recent work it has been shown that the neurogenic deficit in hippocampus can be rescued by physical activity [175, 378]. Lafenetre *et al*. have used a syn-Ras transgenic mouse model where the animal has significantly depressed rates of hippocampal adult neurogenesis that is associated to significant deficit in learning and memory and impaired performance in an object recognition task [378]. Both, impaired neurogenesis and impaired object recognition were rescued by exposing the synRas mice to free access to a running wheel [378]. At the same time the Eisch’s group showed that physical activity could rescue hampered hippocampus neurogenesis induced by knockout of the Notch gene [175].

These data support the hypothesis that continued activity maintains the potential for adult neurogenesis and thereby creates a “neurogenic reserve” capable of sustaining adaptation of the hippocampus network [371].

#### Stress

SGZ hippocampal neurogensis has been shown to be negatively involved in stress reaction [379, 380]. Interestingly, in a social defeat stress paradigm a transient reduction in proliferation rate was observed; on the other hand there were no changes in long-term survival of the newly generated cells. In addition, in a subgroup of mice showing a depressive-like phenotype, there was enhanced neurogenesis in the DG. This neurogenesis is required for induction and maintenance of this special behavior [381].

Stress by environmental noise reduces the proliferation rate of radial astrocytes and neuroblasts. However, these changes do not appear to affect the net number of granular neurons, which suggests a compensatory interaction between cell proliferation and apoptosis. [351]

From these findings it seems that stress caused a transient reduction on neurogenesis that may have no effect on the mature neuronal network. At the same time the neurogenesis is necessary for responses to stress. 

#### Learning

It has been demonstrated that learning impacts on neurogenesis [382], and neurogenesis is involved in learning [383]. Apparently the effects on learning are more pronounced on survival rather than on proliferation of neurons. Interestingly learning tasks that are dependent on the hippocampus, such as trace conditioning and spatial maze training, increase cell survival in this area. On the contrary, delay conditioning [384] and training with a maze, that are hippocampus-independent forms of learning, had no effects on survival of the same cells [385]. Although most of the cells newly generated after the learning task remain in the hippocampus for a period longer than 2 months [386], no clear evidence of their role on modulation of memory has been provided [387, 388]. 

#### Sensorial Stimuli

The effects of sensorial stimuli on neurogenesis have been studied on the olfactory bulb and the spinal cord. 

In the olfactory bulb, GABAergic neurogenesis is enhanced by novel odors [389, 390]. This adaptation is sensitive to the level of the sensory inputs [388]. Experience-dependent synaptic modifications of the newly generated GABAergic interneurons have been described [391]. 

Mechanical sensory stimuli have been shown to induce progenitor cell proliferation in the sensory dorsal (dorsal horn) pathway of the adult spinal cord. The duration of the stimulation, its diversity and adaptation events have significant effects on the neurogenetic response. Neurogenesis in the adult spinal cord has been described [146, 392] but the origin of the proliferating cells induced by mechano-stimulation has not been determined. Interestingly, proliferation was followed by GABAergic neuronal differentiation and increased survival of the newly generated neurons [393].

#### NSC Niches in Aging 

Aging is associated with a decline in hippocampal neurogenesis [394], an event that potentially contributes to age-related dysfunction of the hippocampus. The hippocampus is crucial for learning and memory processes and neurogenesis in this area is sensitive to experience, activity and to age [373, 395]. In aged mice NSC proliferation is dramatically decreased in the SGZ [396]. Interestingly the reduced proliferation rate is not associated by a parallel decrease in NSC density in this niche. This implies that reduced neurogenesis in old mice is not dependent on loss of SGZ NSCs but on their transition to a quiescent state [396]. Interestingly, neurogenesis in old animals is at least in part restored by activity and environmental enrichment [376, 397, 398]. For example after kainic acid injection, proliferation in the aged SGZ is increased to the levels measured in young mice, indicating that the transition to a quiescent state is reversible and that certain stimuli can rejuvenate neurogenesis in the brain [396]. Similar age-dependent decrease in neurogenesis has been shown to occur in the SVZ [399-401]. In this niche the proliferation rate declines by more than 50% in age adults as compared to the young animals. This functional change is accompanied by modification of the SVZ cytoarchitecture [402] indeed the aged SVZ retains a neurogenic dorsolateral zone but it also presents numerous astrocytes penetrating the ependyma and contacting the ventricle. Increased connection with the CSF probably reflects the sensitivity of elderly mice to intraventricular infusion of growth factors [401]. Indeed, by this approach it is possible to stimulate an increase in neurogenesis back to levels found in the young adult animals.

### Modulation by Therapeutic Agents

Several classes of drugs have been reported to interfere with NSC homeostasis. Most of the interest has been focused on psychiatric drugs since the early discovery of links between neurogenesis and stress-related disorders. Several extensive reviews have already been published on this topic in the recent years. Here we focus on clinically relevant drugs that have been shown to interfere with NSC niche homeostasis.

#### Antidepressant Drugs

Association between neurogenesis and depression has been the object of debate since the report of Malberg *et al*. showing in 2000 by BrdU assay that chronic antidepressant treatment significantly increased neurogenesis in the dentate gyrus and hilus of the hippocampus, and that the new cells became neurons [403]. The effects were in agreement with previous data showing that serotoninergic depletion decreased neurogenesis in the DG and the SVZ [404]. The effects on neurogenesis were consistent with the time course for the therapeutic action of antidepressants. Chronic treatment with the classical antidepressant fluoxetine accelerated the maturation of immature neurons and enhanced a specific form of LTP in the dentate gyrus; neurogenesis was also linked to the behavioral effect of fluoxetine measured by the novelty-suppressed feeding test [405]. X-irradiation of a restricted region of mouse brain containing the hippocampus prevented the neurogenic and behavioral effects of antidepressants [406]. Data from human biopsies have also been published using nestin and Ki-67 immunoreactivities as indicators of the presence of dividing neural precursors [407].

There is evidence linking adult neurogenesis to chronic stress, a common etiological factor for anxiety/depressive disorders; and most of the information on depression has been obtained from animal models of depression, including the Unpredictable Chronic Stress Model [408]. Indeed, stress has been shown to be associated to decreased production and survival of newly generated hippocampus neurons [409, 410], whereas treatments with antidepressants increase them and some of the beneficial effects of these drugs require an intact niche [411]. Interestingly, elimination of hippocampal neurogenesis had no effect on animal sensitivity to chronic stress in several behavioral assays, suggesting that reduced neurogenesis is not a cause of stress-related behavioral deficits. Also relevant is that reversal of the stress-induced events by typical antidepressant drugs is only in part dependent on neurogenesis [412, 413] and that recovery from depression by CRF1 or vasopressin 1b antagonists did not require neurogenesis [414]. Inhibition of neurogenesis by antimitotic drugs (rather than ablation of niche by X rays) did not interfere with the antidepressant action of fluoxetine [413]. 

Some of the responses were also dependent on the mice strain used [415] further indicating the complexity of the effects of antidepressants and the difficulties of defining the role of the pro-neurogenic effects of this class of drugs.

#### Antipsychotic Drugs

A more confusing situation is for antipsychotic drugs, as expected by the variety of mechanisms of action of components of this class of drugs. A prominent role in this disorder is played by the dopaminergic system. It must be pointed out that both SVZ and SGZ are innervated by dopaminergic fibers that modulate NSC proliferation probably by activation of D2 receptors [416]. In patients with Parkinson's Disease, SVZ proliferation is markedly reduced [417], a side effect of loss of dopaminergic innervations related to the stimulatory activity of dopamine on EGF and CNTF secretion by SVZ NSCs [418].

Dopamine depletion also inhibits forebrain NSC proliferation [419]. Contrasting data have been reported probably because the effects on NSCs are dependent on several factors, including doses, animal species and brain area since. For example, D2 receptor increased neurogenesis in SVZ in one report [420] and decreased it in another [364]. Similar contrasting effects have been obtained with D3 receptor stimulation [421]. 

Several studies have indicated that some antipsychotics, including haloperidol, clozapine and risperidone, have positive effects on proliferation and survival of neurons from in SVZ and the subgranular zone of the hippocampus. Dawirs *et al* found that treatment of adult rodents with haloperidol increased dentate granule cell proliferation, an effect that maintained the natural spatial septotemporal gradient in granular cell rate of proliferation observed in these animals [422]. On the other hand, neither high doses of clozapine nor haloperidol had any effect on cell proliferation in DG nor did they promote the survival of the newly generated neurons [423]. Altogether the data raise doubts that the therapeutic effects of antipsychotic treatment may be due to the presence of newly generated cells that have migrated to the cortex. On the other hand, glial proliferation occurs in long-term animal treatments with antipsychotic drugs [424, 425] and olanzapine but not haloperidol significantly increased both the total number and density of proliferating non-neuronal cells in the prefrontal cortex and dorsal striatum, whereas no effect was observed in the nucleus accumbens [426]. These observations may explain the increase in thickness of the cortex reported as long/term effect of antipsychotic drugs [424]. Finally, it must be remarked that some of the effects on neurogensis by some of the antipsychotic and/or the antidepressant drugs may be related to interference with production of BDNF and other trophic factor [427].

#### GABAergic Transmission

GABA has important roles on both developmental and adult neurogenesis. The effects vary depending on function of the site of action and on stage of development. In the SVZ as well in the cortex, GABA has a negative role on NSC proliferation and migration [426, 428, 429]. In the adult mouse, application of GABA or its antagonist bicuculline respectively reduced or enhanced the speed of migration of neuronal progenitor cells [424, 425]. The effects of GABA appear to be modulated by astrocytes controlling the extracellular fluid composition surrounding the neuronal progenitor cells. Proliferation of neuronal precursors from the striatum cells is also directly inhibited by GABA receptor activation [424]. 

Accordingly to the major effects of GABAergic stimulation *in vivo*, treatment with diazepam and fluoxetine completely blocked the increase in both neurogenesis and survival induced by the antidepressant [430]. Diazepam also abolished the proliferative effects of a NMDA agonist in adult hippocampus [431].

Phenobarbital and clonazepam significantly inhibited cell proliferation in the dorsal hippocampus and in the dentate gyrus; these effects were apparently related to GABAergic transmission since other anticonvulsant agents were devoid of effects on NSC proliferation [432]. These observations should raise some concern on the use of GABA-based therapies in pregnancy or early in the life. 

Diazepam has opposite effects on hippocampus proliferation in reversal from alcoholic dependence. Using experimental models of alcoholism, it has been shown that alcohol dependence inhibits neurogenesis and that abstinence caused a rebound increase in cell proliferation [433]. Interestingly, the increase in cell proliferation correlated with alcohol withdrawal severity and proliferation remained increased when diazepam [10 mg/kg) was used to reduce withdrawal severity [429].

In this context it is relevant to mention that a number of drugs of abuse have been shown to suppress hippocampal neurogenesis including nicotine, opiates, amphetamines, cocaine and MDMA [434-437]. In all cases, the effects are reversed by withdrawal from drug addiction.

### Remarks from Physiological and Pharmacological Modulation of NSC Proliferation

Proliferation of neural precursors occurs in several physiological and pharmacological experimental models. In most cases the effects are “local” indicating that some degree of selectivity exists in the signals that modulate NSC proliferation in different niches of the brain. Of interest is that the effects of some classes of drugs acting in psychiatric and neurological disorders are mediated or associated to neurogenesis or its inhibition at selective niches. Drugs can therefore be considered tools for studying mechanisms of NSC niche activation; at the same time neurogenesis may be involved in the beneficial effects of some classes of drugs and, perhaps, of non pharmacological therapies. 

Despite clear indication of proliferation, integration of newly formed cells into the pre-existing neuronal networks has not been formally described although functional criteria indicate that this may indeed have occurred. Thus, the question of whether NSC may contribute to brain functional plasticity by adding new neurons to existing networks still remains unanswered. The data in the literature strongly suggest that neurotrophic factors are prominent players in the control of NSC proliferation; since these factors are usually active in close proximity of their sites of release, we propose that in physiological condition modulation of a niche is a local event. In this model, migration from one niche to the other or presence of a stream of NSCs originating from the periventricular zones and directed toward the other niches, appear to be rare events in physiological conditions.

## NEURAL STEM CELL NICHES IN DISEASES

As discussed above, the complex dynamic equilibrium present in healthy adult CNS also involves the participation of functional NSC niches. Conditions that shift the equilibrium are first faced by adaptive responses carried out by both mature and newly formed cells. The occurrence of major events breaking this homeostasis may overcome these adaptive capacities and lead to disease. 

In CNS, various pathogenic events acting by different mechanisms may cause neural cell loss and chronic inflammation. Several agents and mediators sustaining these mechanisms also act on niche homeostasis and it is therefore expected that these two conditions may have a deep impact on NSC biology and NSC niche properties.

The SVZ niche is differentially activated in various neurodegenerative pathologies. An increase in endogenous neurogenesis in the lateral ventricular walls occurs in several diseases including traumatic brain injury, vascular dementia, Huntington’s disease, multiple sclerosis or epilepsy [438-442]. In these conditions, up-regulation of inflammatory cytokine production is a common event. This niche reactivity is maintained even during aging [438]. In several cases it has been shown that the niche reaction leads to generation of new neurons that integrate within the pre-existing circuitry [443]. Appearance of NSC niches at unexpected ectopic sites also occurs in brain parenchyma following stroke. In that case, part of the ectopic DCX pool induced by stroke originates from the SVZ-resident NSCs that have migrated to the injured site [444]. In other cases this ectopic pool may originate locally [445, 446], leading to an injury-induced ectopic NSC niche formation. Niche reaction to injury may thus proceed in three ways: *i)* amplification of pre-existing NSC niche and migration toward the damage site; *ii)* activation of local and pre-existing quiescent NSC niches; *iii)* conversion of a normal tissue into a newly established NSC niche. The discovery of a “new” niche envisaged in *iii* may simply reflect lack of detection of a quiescent niche in normal condition.

The mechanisms and signals driving these reactions are many, some of them peculiar to the pathological condition and most of them endogenous to the NSC niche and already acting in physiological condition. 

In the following paragraph we briefly discuss some examples of injury-induced NSC niche activation. Before reviewing the hallmarks of the involvement of NSCs in selective diseases, we will first consider how some of the extrinsic factors involved in niche homeostasis may contribute to this activation. 

### Signals in Diseases

Brain abscess, trauma, multiple sclerosis, diabetic ketoacidosis and stroke alter BBB function, thus causing life threatening oedema. In this condition the plasma component fibrinogen is released into the brain tissue leading to secretion of TGF-β, the starting signal for reactive gliosis [317]. This phenomenon includes astrocytic hypertrophy and upregulation of intermediate filaments, including nestin, loss of the polarized expression of proteins of astrocytic endfeet and secretion of chondroitin sulphate proteoglycans (CSPGs). The new instructive cues appear to originate mainly from the inflammatory reaction, that includes vasodilation and cytokine secretion, and from disruption of the cytoarchitecture of the tissue that involves signalling from the basal lamina. Inflammation is a critical response that may lead to repair or elimination of the tissue. In CNS, inflammatory reaction aims at limiting and engulfing the damage rather than seeking repair. The inflammatory reaction involves primarily the microglia, i.e. the resident macrophage population in the parenchyma, attracted to the site of injury by chemotactic factors [447] and acting as phagocytes and antigen presenting cells [448]. Activated microglia secretes many inflammatory cytokines that recruit blood-born leucocytes and amplify the inflammatory reaction [449]. At the site of injury, the infiltration of monocytes increases the level of SDF-1, the prominent chemoattractant for lymphocytes and monocytes [450]. In stroke animal models, SDF-1 is increased and released primarily by activated astrocytes and endothelial cells [451]. 

The gradient of SDF-1 in this condition induces NSC exit from the SVZ niche and migration to the site of injury [452]. Moreover, locally produced SDF-1 may attract other cells, including endothelial progenitor or meningeal cells, further increasing the complexity of the injured microenvironment. The vasodilatation that occurs in inflammatory process can alter tissue oxygen tension at site of injury. Hyperoxia is known to trigger NSC proliferation/differentiation [242, 243, 453]. On the other hand, injury conditions where oxygen tension decreases, such as stroke or tumors, may induce a hypoxic adaptation involving several factors including HIF-1α and HIF-2α. These signals can upregulate stem-related genes such as Oct4 [454] and Notch target genes [235, 455]. Thus, in a pathological context the possible influence of changes in oxygen tension on the generation of ectopic injury-induced NSC niches should be taken into account. 

Changes in ECM components may also be a signal for NSC niche activation in disease. As discussed above, several ECM components such as CSPG, tenascin, hyaluronan and hyaluronan-binding proteins (heparansulfate or agrin) modulate proliferation and differentiation potentials in the niche by binding to the toll-like receptors TLR-2 and -4 that are expressed in some of the NSCs [456]. Changes in ECM components are usually associated to diseases, as in the case of CSPG that is upregulated after spinal cord injury [456]. 

It must be noted that perpetuation of “dangerous signals” occurring in chronic inflammation appears to decrease NSC proliferation in SVZ [457]. This modulation of the response of the endogenous brain NSC compartment appears to be mediated by Th1 cytokines patterns, including high level of IL-1, INF-gamma and TNF-alpha [457]. 

These pathogenetic mechanisms are shared in most of the CNS diseases; however involvement of individual components of the reactive mechanisms may vary depending on the condition and location. Therefore reaction(s) of NSC niches to pathological condition should not be expected to be homogeneous.

### NSC Niches in Pathology

#### Stroke

Ischemia and reperfusion cause dramatic variations in parenchymal microenvironment, including O_2_ concentration, depletion of ATP, perturbation of the cellular ionic homeostasis, inflammation and abnormal neurotransmitter release [458]. Reduction of cerebral blood flow leads to a lack of oxygen and glucose supply to the brain parenchyma. This implies that even short changes of blood flow in the brain can have profound effects on NSC proliferation and migration [459]. Indeed, ischemic stroke has been shown to induce proliferation of endogenous NSCs and to increase the number of immature neurons in the SVZ of the adult rodent brain [58, 460-463] as well as the migration of DCX-neuroblasts to the peri-infarct regions [220, 444, 452, 464, 465]. This migration of neuroblasts runs from the SVZ and SGZ to the peri-infarct cortex and striatum and follows blood vessels [444]. These neuroblasts have the potential to differentiate into mature neurons [452, 466 467]. Generation of new cortical neurons induced by cerebral ischemia has been shown to occur in the human brain [468]. 

Signals that stimulate stroke-induced neurogenesis within the SVZ have not been fully characterised. It is known that TNF-alpha possesses proangiogenic and proneurogenic effects [308] and indeed the SVZ-NSC proliferation induced by stroke requires the TNF-α converting enzyme protease activity [308]. Excitotoxicity, a major cytotoxic mechanism in the early stages of the progression of ischemic brain injury, is also expected to interfere with neural precursor homeostasis [469].

As reported above, variation of O_2_ concentration occurring in ischemia influences NSC proliferation and differentiation [237-244]. It must be pointed out that oxygen/glucose deprivation may influence other resident niche cells as well, including endothelial cells, which may differentially modulate NSCs [470, 471]. Finally, injured cerebral endothelium allows peripheral leukocyte invasion within hours after the onset of focal cerebral ischemia. These cells, together with activated astrocytes and microglia, secrete a large variety of cytotoxic agents [472]

Altogether these events may interfere with the survival of the newly generated NSC progeny and need to be taken into account when designing a stem cell based therapy for stroke. 

#### Multiple Sclerosis

Contrasting reports have been published on the state of activation of the SVZ niche in multiple sclerosis (MS). Indeed, an increase in NSC and/or OPC proliferation (to some extent associated to oligodendrogenesis) has been observed in SVZ both in human subjects and in animal models of multiple sclerosis [473]. Oligodendrocyte progenitors are present in MS lesions in significant numbers [474-476] and spontaneous myelin repair has been observed [477], an event that appears to be more widespread and successful than expected [478].

Other authors have shown a decrease in quiescence and in transient amplifying cells in SVZ with an increase of neuroblasts density [457]. These observations suggest that the inflamed brain microenvironment sustains alteration of the NSC niche [457, 464].

#### Parkinson’s Disease

Parkinson’s disease (PD) is caused by a progressive degeneration of the midbrain dopamine neurons in the substantia nigra pars compacta that predominantly affects the ventral population projecting to the dorsal striatum and leads to a gradual dysfunction of the motor system. PD is generally associated with an inflammatory reaction characterized by the presence of activated microglia and elevated serum or cerebrospinal fluid levels of pro-inflammatory factors such as TNF-α, nitric oxide and IL-1β [479]. Dopamine depletion and neuro-inflammation lead to a decrease numbers of proliferating cells in the subependymal zone and neural precursor cells in the subgranular zone and olfactory bulb detected in both animal models and postmortem brains of individuals with Parkinson disease [480]. Proliferation of transit-amplifying C cells in the SVZ is restored completely by DA agonists (ropinirole and pramipexole) and selective agonist of D2 or 3 receptors [481]. In PD neurogenesis is depressed by a mechanism that also involves reduced secretion of EGF and CNTF by NSCs caused by the decreased dopaminergic transmission [260, 418]. 

Inflammatory responses in the substantia nigra and striatum are thought to be responsible for the progression of PD [482]. Interestingly, use of drugs with anti-inflammatory activity prevents dopaminergic neurodegeneration in experimental models of PD [483]

#### Spinal Cord Injury

Following mechanical injury (SCI), the normally limited proliferation of ependymal cells is increased, followed by migration of the ependyma-derived progeny toward the site of injury. Under this condition, at least a sub-population of the spinal cord ependymal cells displays latent neural stem cell properties [147]. Ependymal cells give rise to a substantial proportion of the scar-forming astrocytes as well as to rare oligodendrocytes [49]. These newly formed oligodendrocytes likely contribute to remyelinization [484].

In addition, nestin-positive cells not originating from the central canal have also been observed in injured spinal cords [49] and neurogenesis involving DCX-positive precursors of the spinal cord promotes functional recovery from injury [485]

Apart from ependymal cells, it has been shown that adult spinal cord NSCs are responsive to injury. The SCI-induced response includes division, migration, and maturation of SC progenitor cells [486]. A time course study of a contusive SCI in adult rodents has shown that proliferation of cells expressing NG2 reaches a peak at 3 days, stays markedly increased in the epicentre of the lesion, and declines to baseline levels by 4 wk after injury. 

Our data indicate that also the nestin-positive cells present in the leptomeninges of the spinal cord proliferate in response to moderate SCI [167]. These cells, that form neurospheres *in vitro* and can be differentiated by trophic factors in functional neurons or in mature oligodendrocytes, also mature and form DCX/positive neuroblasts. By using a lentivirus-labelling approach, we have shown that meningeal cells, including nestin- and doublecortin-positive cells, migrate in the spinal cord parenchyma and contribute to the glial scar formation [167].

Inflammation induced by SCI is another important player in spinal cord progenitor cell induction by pro-inflammatory cytokines and peripheral immune cells. Indeed, following CNS injury, an intensive local inflammatory response takes place. This reaction involves activated resident microglia and monocytes which infiltrate the CNS from the peripheral blood as consequence of the traumatic damage [487]. Blood-born macrophages in injured CNS have been shown to be a functionally heterogeneous population [145, 312, 488]; they promote removal of tissue debris [489, 490], are capable of secreting neurotrophic factors [491], and can support axonal regeneration [492]. Interestingly, macrophages pre-incubated *ex-vivo* with peripheral nerve segments can induce partial recovery of paraplegic rats when implanted *in vivo* [493] 

Depending on whether the microglial cells are stimulated by IFN-α released by T helper 1 (Th1] cells or by IL-4 from Th2 cells, they differentially influence the fate choice of spinal cord progenitor cells: microglia stimulated with IL-4 protect oligodendrocyte progenitors [494], whereas microglia stimulated with IFN-α preferentially induced neurogenesis [495]. 

These data highlight the complexity of the reaction to inflammation and support the notion that the responses and the fate of individual NSC niches are strictly dependent on the pre-existing characteristics of the niche microenvironment and on the intensity, evolution and properties of the locally active inflammatory response. 

This complexity also indicates that insights on the physiological function of NSCs and/or their niche will be difficult to be obtained or extrapolated from pathological conditions.

#### NSC Niche for Neural Cancer Stem Cells

The presence of cancer stem cells has been demonstrated in several high-grade primary brain tumors, including gliomas, medulloblastomas, ependymomas and neuroblastomas. Cancer stem cells in neural cancers display similarities with NSCs in terms of capacity for self-renewal, sphere-forming growth in serum-free conditions, stem cell marker expression and multilineage differentiation potential. Recently many insights into the mechanisms of growth-sustaining communication between cancer stem cells and the microenvironment have been revealed and shown to share common mechanisms with NSC niche [496]. Understanding of how quiescent cancer stem cells interact with their microenvironment has direct implications on the basic biology of the tumor and is likely to provide insight into the design of more effective therapies. Cancer and niche common key extrinsic regulations involving the microenvironment have been described including hypoxia [497], interaction with vasculature and perivascular structures [498] and with ECM [499].

## CONCLUDINGS REMARKS

Presence of neural stem cells in adult mammalian brains, including human, has been clearly demonstrated by several studies. The functional significance of adult neurogenesis is slowly emerging as new data indicate the sensitivity of this event to several “every day” external stimuli such as physical activity, learning, enriched environment, aging, stress and drugs. In addition, neurogenesis appears to be instrumental for task performance involving complex cognitive functions [378]. 

Despite the growing body of evidence on the functional significance of NSC in physiological events and in pathologic condition, and despite the growing mass of data concerning the molecular and cellular properties of NSCs and their niches, several critical questions are still open.

First is the identification of the stem cells themselves and of their origin. 

A unique “identity card” of NSC is still lacking. This is probably because NSCs are not static entities and are continuously changing phenotype in response to environmental clues. A critical example is the ability of DCX-positive neuroblasts to differentiate into either neurons or oligodendrocytes depending on the environmental clues to which they are exposed.

In recent years introduction of new transgenic animal models has allowed genetic-fate mapping *in vivo*. Results of these studies are very informative but they are dependent on the transgenic model used. Moreover, the identification of the *in vivo* fate of a precursor cell allows identification of the original NSC clone but not of its original location. Thus the question of the source of NSC in brain is still open.

Proliferation in the SVZ has allowed the first recognition of neurogenesis in adult brain. Since then, many other NSC niches has been found and characterized in the adult brain. In addition, intermediates of neurogenesis as the neuroblasts are present and spread in various areas of the CNS, including the subpial zone, the cortical layer II, the hypothalamic region, and the dorsal horn of the spinal cord parenchyma. These multiple locations increase even more when injury related signals activate the “*bona fide*” NSC niche and/or the injury-induced ectopic NSC niche. 

The second critical question concerns the mechanisms of delivery of newly generated cells to their final destination. This is a potential problem because the NSC niches, despite their growing number, are still limited. Even when neurogenesis appears to be a local event, as for the SGZ niche that is in tight relation with hippocampus function, migration from the niche to the site of integration remains an obscure event about which information are lacking. Insights on the travelling mechanisms may derive from deeper studies on the biology of the SVZ NSCs that are sources of olfactory bulb interneurons and of neuroblasts migrating to ischemic regions. In the first case migration occurs through the rostral migratory stream, in the second perivascular tracks are supposed to guide migration. 

In this respect meninges may be a strategic player due to their high anatomical complexity, widespread distribution and intimate connection with the CNS parenchyma. Indeed, meninges not only cover the entire CNS; they are also widely spread inside the parenchyma as they follow vessels organizing the perivascular space. The meninges are thus a net of potential trails properly suited to allow migration of precursor cells toward any site within the CNS.

Meninges are also endowed of many of the components known to play crucial role in NSC niches including the presence of well characterized neural precursors. This observation, as well the diffused presence in the cortex of neuroblasts, raises the question of whether *plasticity by addition* of new cells requires newly generated cells. Indeed, meninges can be considered both as a large and extended niche hosting precursors that can be delivered on demand to adjacent sites or as a trail allowing transit of precursor to distant sites. In this view, all the NSC niches in the CNS may be connected in a functional network using the threads of the meningeal net as tracks Fig. (**4**). 

If this is the case, proliferation of NSC may not be considered the hallmark of niche activation. Or, at the least, proliferation may occur at niches that are located very distant from the region where newly generated neurons or glial cells will integrate in the tissue. 

This challenging hypothesis that we provide significantly changes the biological significance of meninges for the functioning of the brain. 

## Figures and Tables

**Fig. (1):**
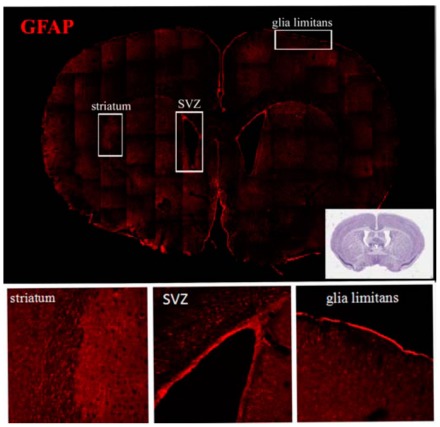
**Distribution of GFAP immunoreactivity in adult rat brain** GFAP expression is visualized by immunofluorescence using chicken anti-GFAP antibodies (Abcam, dil. 1: 1000). The image is reconstructed from collection of high-resolution confocal microscopy images. Boxes show high magnifications of the regions with highest levels of immunoreactivity: striatum, lateral ventricular zone and the glia limitans. Bright field histological section stained by H&E.

**Fig. (2):**
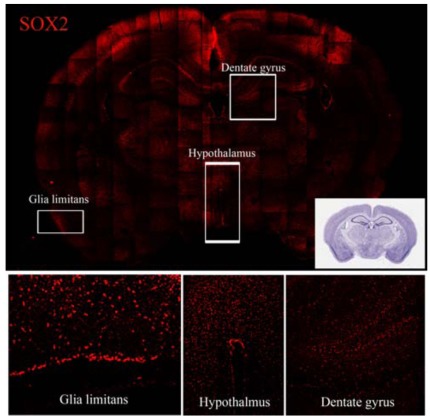
**Distribution of SOX2 immunoreactivity in adult rat brain Map** of SOX2 expression (red) in adult rat brain by anti-Sox2 goat antibodies (Santa Cruz, dil. 1: 1000]. Image reconstructed from collection of high-resolution confocal microscopy images. Boxes show high magnification of dentate gyrus of the hippocampus and hypothalamus, external border of the cortex.

**Fig. (3):**
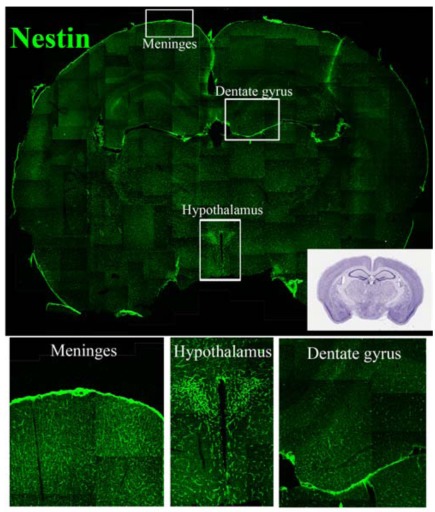
**Distribution of nestin immunoreactivity in adult rat brain** Map Nestin expression in adult rat brain visualized by mouse anti-rat Nestin antibodies (BD Bioscience, dil. 1: 1000] (green): As for Figs. (**1** and **2**), this image is reconstructed from collection of high-resolution confocal microscopy images. Nestin distribution shows regions with high expression levels of this NSC marker. Note the frequent association of nestin immunoreactivity with *bona fide* vessels of the parenchyma. Boxes show high magnifications of lateral ventricular zone, external border of the cortex, dentate gyrus of the hippocampus and hypothalamus.

**Fig. (4):**
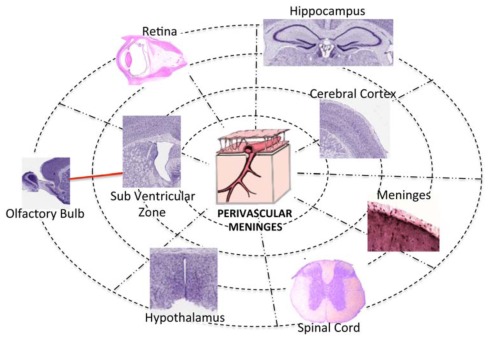
**NSC niche network hypothesis** NSC niches have been described in many region of the adult brain, including sub ventricular zone, hippocampus, cerebral cortex, olfactory bulb, retina, spinal cord and meninges. The niches may exist as individual entities or as a network of niches. Data on responses of niches to physiological, pharmacological and pathological stimulation suggest that niches form a network, possibly centered on the SVZ that may act as the “reservoir” of the brain NSCs. The red lines highlight well established connections between niches. Meninges may have a dual role: they are both one of the niches of the network and the anatomical branches of the net sustaining connection between niches and NSC migration to sites of integration into the normal tissue.
